# A novel theta-controlled vibrotactile brain–computer interface to treat chronic pain: a pilot study

**DOI:** 10.1038/s41598-024-53261-3

**Published:** 2024-02-10

**Authors:** Phillip Demarest, Nabi Rustamov, James Swift, Tao Xie, Markus Adamek, Hohyun Cho, Elizabeth Wilson, Zhuangyu Han, Alexander Belsten, Nicholas Luczak, Peter Brunner, Simon Haroutounian, Eric C. Leuthardt

**Affiliations:** 1 Division of Neurotechnology, Department of Neurosurgery, Washington University in St. Louis School of Medicine, St Louis, MO 63110 USA; 2 Department of Biomedical Engineering, McKelvey School of Engineering, Washington University in St. Louis, St Louis, MO 63130 USA; 3 Department of Neurosurgery, Washington University in St. Louis School of Medicine, St Louis, MO 63110 USA; 4 Division of Clinical and Translational Research, Department of Anesthesiology, Washington University in St. Louis School of Medicine, St Louis, MO 63110 USA; 5 Washington University Pain Center, Washington University in St. Louis School of Medicine, St Louis, MO 63110 USA

**Keywords:** Chronic pain, Outcomes research

## Abstract

Limitations in chronic pain therapies necessitate novel interventions that are effective, accessible, and safe. Brain–computer interfaces (BCIs) provide a promising modality for targeting neuropathology underlying chronic pain by converting recorded neural activity into perceivable outputs. Recent evidence suggests that increased frontal theta power (4–7 Hz) reflects pain relief from chronic and acute pain. Further studies have suggested that vibrotactile stimulation decreases pain intensity in experimental and clinical models. This longitudinal, non-randomized, open-label pilot study's objective was to reinforce frontal theta activity in six patients with chronic upper extremity pain using a novel vibrotactile neurofeedback BCI system. Patients increased their BCI performance, reflecting thought-driven control of neurofeedback, and showed a significant decrease in pain severity (1.29 ± 0.25 MAD, p = 0.03, q = 0.05) and pain interference (1.79 ± 1.10 MAD p = 0.03, q = 0.05) scores without any adverse events. Pain relief significantly correlated with frontal theta modulation. These findings highlight the potential of BCI-mediated cortico-sensory coupling of frontal theta with vibrotactile stimulation for alleviating chronic pain.

## Introduction

Currently, available approaches for treating chronic pain have limited therapeutic benefits and are often associated with side effects. Only 30–40% of patients with chronic pain achieve meaningful pain relief with pharmacotherapy^1,2^. Many of these patients receive opioids, which are associated with increased risks of dependence and substance use disorders^3,4^. Invasive approaches for treating chronic pain, such as deep-brain stimulation and spinal cord stimulation, are effective in some patients but are expensive and can lead to complications^5,6^. Given the current limitations in treating chronic pain, there is a critical need to develop novel, safe, non-invasive, and affordable therapies.

Chronic pain is associated with pathological changes in neural circuitry, including maladaptive neuroplasticity, cortical reorganization, and changes to descending pain modulation pathways^7–10^. In humans, brain–computer interface (BCI) systems have emerged as a promising tool for remodeling neural circuits^11^. This premise of BCI relies on Hebbian learning—i.e., activity-dependent synaptic plasticity where concurrent neuronal activations strengthen neural connections. BCIs can decode cortical physiology in real-time and provide temporally precise outputs to enable the necessary conditions for Hebbian learning^12^. This physiologic and temporal precision enables powerful conditions for enhancing neuroplastic changes. Specifically, BCIs enable robust *cortico-sensory coupling,* where a desired brain physiology is amplified with functionally relevant sensory feedback that is provided in real-time and with high temporal precision^13^. This BCI-mediated method has enabled novel therapies for what have been considered intractable neurologic conditions, such as chronic stroke-induced hemiparesis^14–16^. Early efforts in using BCI as a pain intervention have been attempted with mixed results and rarely report relationships between physiology and outcome^17–20^. This variable effect is partly due to the brain signals used and the feedback provided. Specifically, past BCIs have commonly used cortical sensorimotor rhythms associated with motor imagery and visual feedback^17,18,20–22^. Neither of these inputs and outputs are directly relevant to pain perception. Thus, in the setting of chronic pain, it will be essential to couple cortical signals associated with pain relief with pain-alleviating sensory input, such as vibrotactile stimulation^23–25^, to best enable neural remodeling to occur. Further, the variation in brain regions previously reported in pain processing justifies targeted reinforcement of physiology consistently observed in multiple contexts^26^. The current work builds on the previous demonstrations that relief of both chronic pain and experimental acute pain is associated with increased frontal theta (θ) rhythms (4–7 Hz)^27,28^.

Increases in frontal θ have been previously reported during cognitive tasks involving attention and concentration (such as working memory tasks, mental arithmetic, and meditation)^29–32^. Some of these tasks have previously been shown to have implications for pain management, such as the positive clinical outcomes associated with meditation or the phenomenon of distraction-induced analgesia^32–37^. Recent reviews of studies that have elucidated the neural correlates of acute and chronic pain have revealed divergent evidence. Studies that reported changes in θ observed variable results, with some reporting increases, decreases, or no changes. The locations of reported changes in θ were also highly variable, with most studies only reporting changes in global activity^38,39^. Further, these studies are primarily associative and are unable to decouple biomarkers of pathophysiology (i.e., active contributors of pathology) from biomarkers of homeostatic responses (i.e., compensatory mechanisms that reduce pathology). This limitation of previous studies prevents inferences about how a particular EEG activity *contributes* to symptoms of pain and only provides evidence of co-occurrence. As such, approaches that reinforce pain-related biomarkers and subsequently observe their effect on pain-related symptoms are crucial for interpreting the relationship between EEG features and pain symptoms.

To this end, we created a novel BCI system that is controlled by frontal θ rhythms and uses vibrotactile stimulation of the affected area as a means of neurofeedback in patients with medically refractory chronic upper extremity pain. In order to justify placebo-controlled clinical trials that leverage this novel electrophysiological target for neuromodulation in the future, the present study aimed to test the feasibility of BCI-mediated reinforcement of frontal θ rhythms, and whether this physiology can be linked to symptom relief in chronic pain patients. Furthermore, using secondary analysis, we aimed to identify mechanistic evidence related to BCI therapy that warrants future elucidation and study design refinement. We hypothesized that a longitudinal intervention with such a BCI-controlled vibrotactile system would be feasible, safe, and would reduce pain severity and pain interference as measured by the Brief Pain Inventory (BPI). Therefore, the objective of this pilot trial was to test the feasibility and determine the initial efficacy of treating chronic upper extremity pain with a BCI-controlled vibrotactile system and identify potential neurophysiological correlates of the observed clinical outcomes. Taken together, the findings in this work provide early compelling evidence that BCI-mediated cortico-sensory coupling may provide a novel approach to alleviating chronic pain in the upper extremity and justify conducting randomized, controlled clinical trials in the future.

## Results

### Patient characteristics

This study included six patients diagnosed with upper extremity chronic pain who met the inclusion criteria (3 females, three males; age: 59.5 ± 5.5 [median ± median absolute deviation (MAD)]; range 18–74 years). The median pre-BCI intervention Visual Analog Scale (VAS) pain rating was 47.5 (± 18.5 MAD). Patient demographics, characteristics, and comorbidities are shown in Table 1. Participant Hospital Anxiety and Depression Scale (HADS) score was 5.5 (± 2.0 MAD) for depression and 5.00 (± 2.50 MAD) for anxiety. Two of the six patients had depression and anxiety scores above the normal 0–7 range. The total score for Pain Catastrophizing Scale (PCS) was 19.5 (± 13.0 MAD). Table 2 shows the HADS and PCS results for all patients. Four of the six patients had hypersensitivity in their affected area. One had heat and pinprick hypersensitivity, two had pinprick hypersensitivity, and one had brush and heat hypersensitivity. The location of the pain area and type of hypersensitivity for each patient are shown in Supp. Fig. 1. Three of the patient’s affected areas were bilateral, with the average reported pain intensity being the same on both sides. Hypersensitivity, Quantitative Sensory Testing (QST), and conditioned pain modulation (CPM) tests were conducted on the affected side with greater pain intensity on the day of the baseline visit. The results of QST and CPM are reported in Supp. Tables 1 and 2, respectively. The baseline median scores on the BPI, the primary outcome measure, were 5.25 (± 1.63 MAD) for the Pain Severity Score (PSS), and 4.86 (± 2.29 MAD) for Pain Interference Score (PIS). The median Neuropathic Pain Symptom Inventory (NPSI) total score before BCI intervention was 17.9 (± 9.60 MAD).

**Table 1:** Patient demographics and medical history.

Patient	Age	Sex	Race	BMI	Duration of chronic pain (years)	Previous interventions	Diagnosis/medical history	Pain medications	Non-pain medications
1	60	M	Caucasian	33.74	0.5	Physical therapy, gabapentin	D: cervical radiculopathy MH: type II diabetes, hypertension, high cholesterol, herniated disc in thoracic area	Hydrocodone-acetaminophen	Lisinopril, fish oil, fluticasone, atorvastatin, metformin, gabapentin
2	50	F	Black/African	45.2	3.0	Physical therapy, chiropractor, NSAIDs		Oxycodone, acetaminophen	Albuterol, mometasone/formoterol, gabapentin, vitamin D
3	18	F	Caucasian	22.3	1.5	Local, corticosteroid, injections, occupational, therapy, acupuncture, NSAIDs	D: tenosynovitis 1st dorsal compartment	Ibuprofen, acetaminophen	
4	74	M	Caucasian	27.5	20+	Physical therapy, NSAIDs	D: scapholunate advanced collapse MH: arthritis, obstructive sleep apnea, hyperlipidemia	Ibuprofen, acetaminophen	Simvastatin, allopurinol, potassium citrate, tadalafil, zolpidem, sildenafil, valaciclovir, daily multivitamin
5	61	F	Caucasian	37.2	20+	Chiropractor, platelet rich plasma therapy, physical therapy, NSAIDs	D: cervical radiculopathy MH: hypothyroidism, head trauma	Celecoxib	Levothyroxine
6	59	M	Caucasian	23.4	0.4	N/A	D: post-surgical nerve injury MH: laminectomy fusion, syndrome, cervicalgia, cervical radiculopathy, neck pain, hyperlipidemia	Gabapentin, duloxetine, pregabalin	

*BMI* body mass index, *M* male, *F* female, *NSAIDs* nonsteroidal anti-inflammatory drug, *D* diagnosis, *MH* medical history.

**Table 2:** Patient HADS and pain catastrophizing scale scores.

Subject	HADS		Pain Catastrophizing Scale		Helplessness	Total
	Depression	Anxiety	Ruminition	Magnification		
1	6	3	3	0	12	15
2	15	15	16	11	22	49
3	3	6	7	2	15	24
4	5	4	4	1	4	9
5	4	3	2	0	2	4
6	10	9	15	2	18	38

*HADS* Hospital Anxiety and Depression Scale.

### Intervention retention, adherence, safety, and comfort

**Figure 1:**
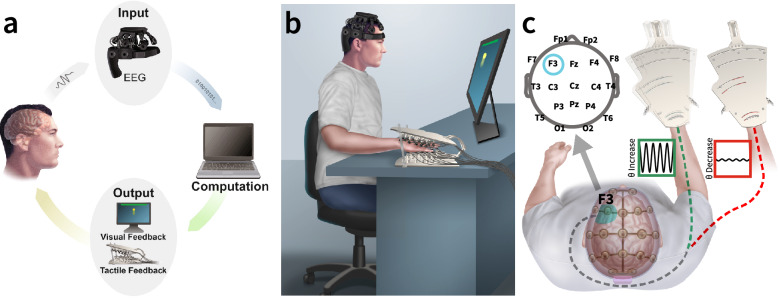
Schematic and description of BCI system. (**a**) System overview schematic of EEG-driven BCI system consisting of EEG signals as input and dual visual and vibrotactile sensory feedback. Electrophysiological activity is recorded using a 24-channel dry EEG headset connected to a PC laptop via Bluetooth. The PC uses BCI2000 for real-time signal processing (i.e., extrapolating frontal midline θ power changes) and command execution (i.e., initiating visual and tactile feedback in response to changes in frontal θ power). Visual feedback was delivered on an external monitor, and tactile feedback was delivered using the custom HVSA. (**b**) System set up with BCI system with vibrotactile feedback system enclosing the affected hand. (**c**) Overview of electrode configuration (Top-left) and vibrotactile neurofeedback. Increased F3 θ modulation (teal) during relevant phases of BCI therapy leads to vibrotactile feedback of the affected area (green). No modulation of θ or decreased modulation of θ leads to no vibrotactile feedback (red).

Patients underwent three to five one-half to 1-h BCI training sessions per week over 5–6 weeks. The intervention duration was specified to be at least 5 weeks and no more than 6 weeks. The BCI system consisted of a dry electrode electroencephalogram (EEG) headset, a laptop, a custom-made hand vibration stimulation array (HVSA) for functionally relevant sensory feedback, and a video screen for visual feedback (Fig. 1a,b). Figure 1c shows all electrode locations used during recordings, and the integration of the EEG system and the HVSA, where vibrotactile stimulation is delivered to the affected area as a function of patient-generated frontal θ power.

**Figure 2:**
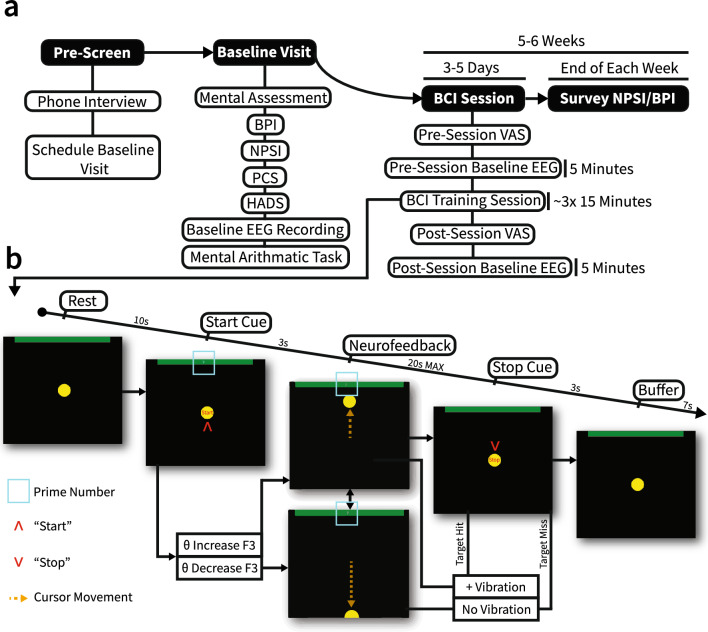
Overview of BCI therapy. (**a**) Overall treatment timeline. Solid black shapes indicate the main therapy sections. White shapes indicate specific breakdowns of each main section. (**b**) Overview of a single BCI trial. The teal box shows the numerical stimulus used in early BCI sessions to induce θ modulation. “Start” and “Stop” cues are indicated by red arrows. Cursor movement is indicated by the orange dotted arrow. Trial states progress from left to right. θ modulation direction leads to movement of the cursor during neurofeedback. The neurofeedback period was the only period with a variable duration, with 20 s being the maximum duration of the neurofeedback. If the cursor reaches the green target at the top of the screen, several pulses of vibration are delivered to the affected area over the duration of the “Stop” cue.

The overall design of the intervention is shown in Fig. 2a. Patients meeting inclusion and exclusion criteria attended an initial baseline visit to determine their pain symptom characteristics, record baseline EEG, and perform an initial assessment of task-mediated frontal θ modulation (Supplemental Methods)^30,38^. VAS pain scores were obtained before and after BCI training (main intervention) in each of these sessions. BPI, consisting of pain severity score and pain interference score (PSS and PIS, respectively), and NPSI metrics were obtained once per week via survey after all of each week’s training sessions. A specific breakdown of the structure of BCI training sessions is shown in Fig. 2b.

**Figure 3:**
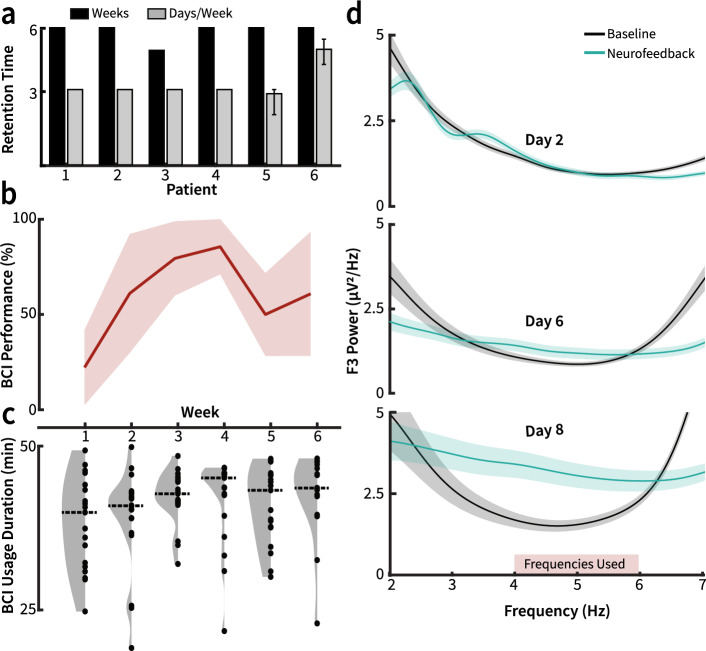
Intervention feasibility. (**a**) Each patient’s intervention time. Black bars represent the number of weeks each patient underwent BCI training. Gray bars represent the mean number of BCI training days per week. Error bars represent the range of days per week completed. All patients underwent at least 3 days of BCI training per week for 5 weeks. Four of the patients completed 3 days of BCI training every week. One patient completed 3 days of training per week for 5 weeks, then completed 2 days of training on the sixth week (range 2–3, where only two sessions were completed on the sixth week). One patient completed 4–5 days of training per week for 6 weeks (range 4–5). (**b**) Median BCI performance across all participants across 6 weeks of BCI therapy. The shaded region depicts the median absolute deviation. (**c**) Violin plots reflecting session duration across all patients each week. The dotted line represents the median session time across all patients each week. There was no significant difference in session duration between weeks (n = 19, 20, 19, 19, 19, 16; Kruskal–Wallis test, df = 5, χ^2^ = 8.28, p = 0.14). (**d**) Exemplar power spectral density plots comparing average θ range power during rest and neurofeedback phases of BCI training for patient 1. Shaded regions represent standard error. θ modulation increases over BCI training sessions. The red rectangle indicates frequencies used for BCI real-time signal processing.

A total of eight patients meeting the inclusion/exclusion criteria attended an initial baseline visit. One patient could not meet the retention criteria and withdrew from the study after the baseline visit (recruitment rate 87.5%, 7/8). One other patient could not adhere to the treatment regimen due to scheduling conflicts and withdrew from the study after the fourth week (retention 85.7%, 6/7). The regimens followed by each of the six patients who completed the pilot study are visualized in Fig. 3a. Five out of the six patients completed 6 weeks of BCI intervention. One patient completed 5 weeks. The retention and recruitment rates of this study are comparable to the rates found in larger-scale randomized controlled trials (median recruitment rate 72%, median retention rate 88%)^40^ and comparable studies using longitudinal therapeutic BCIs (recruitment range 68.4–80.5%, retention range 55.6–76.9%)^15,21,41^.

The median BCI performance across all 6 weeks of treatment is shown in Fig. 3b. Patients underwent a median BCI training session duration of 43 min, and completed a median of 1,192 BCI trials over the intervention period. There were no significant changes in weekly session duration throughout the pilot (n = 19, 20, 19, 19, 19, 16; Kruskal–Wallis test, df = 5, χ^2^ = 8.28, p = 0.14; Fig. 3c). Each patient’s daily session duration is visualized in Supp. Fig. 2. The total number of trials each patient underwent during each BCI training day and across the entirety of the intervention is shown in Supp. Table 3. Use of the BCI system did not lead to any apparent complications or discomfort for any of the patients. No patients reported any issues or additional symptoms throughout the course of the intervention.

### Feasibility of frontal theta modulation

To confirm the feasibility of our intervention in creating observable differences in task-mediated frontal (F3) θ power, patients performed a serial subtraction mental arithmetic task during their baseline visit, and differences in power spectra were analyzed. The results from this task are shown in Supp. Fig. 2. These data demonstrate that the serial subtraction task can create qualitative differences in θ power in patients; however, the effect sizes, specificity, and directionality vary. Figure 3d shows an exemplar progression of power spectral density in θ range for a single patient during BCI training for neurofeedback and rest periods at the F3 electrode. There is a clear qualitative separation of power in θ range between the neurofeedback period and baseline (rest) period of BCI training. Furthermore, the magnitude of the θ modulation during neurofeedback increases with each exemplar BCI training session. The comparison of differences in θ power between the pre-intervention baseline and BCI intervention is shown in Supp. Fig. 2. The normalized θ power change over each BCI session neurofeedback period across all patients is shown in Supp. Fig. 2. In sum, these results reflect the feasibility of our pre-screening task and BCI intervention in producing and detecting modulation in frontal (F3) θ power.

### Clinical outcomes and BCI performance

**Figure 4:**
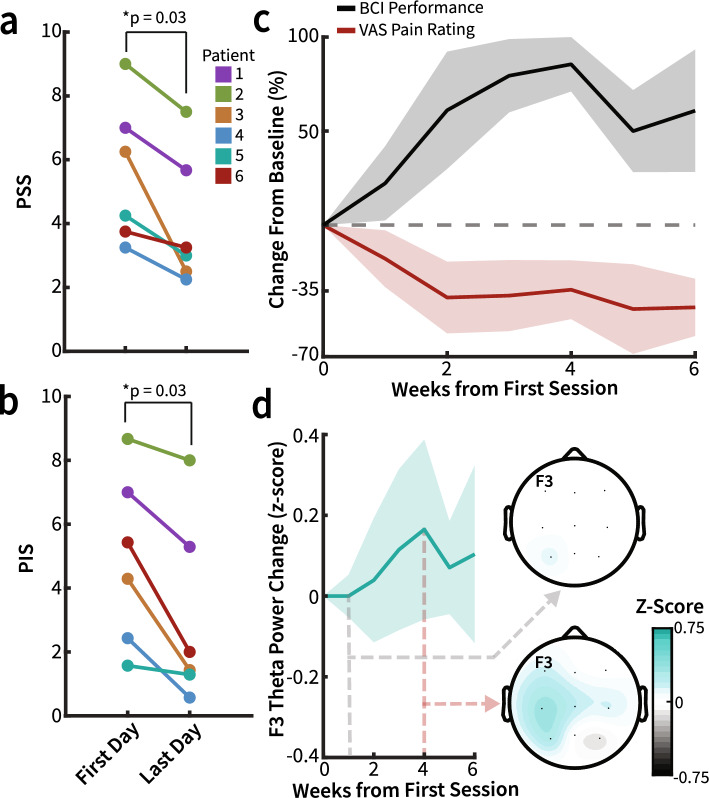
Vibrotactile BCI therapy decreases chronic pain symptoms. Individual patient outcomes: (**a**) PSS (N = 6, Wilcoxon signed-rank, median decrease 1.29 ± 0.25 MAD p = 0.03, q = 0.05) and (**b**) PIS (N = 6, Wilcoxon signed-rank, median decrease 1.79 ± 1.10 MAD p = 0.03, q = 0.05) significantly decreases after the BCI intervention duration. (**c**) Median BCI performance and VAS rating for all participants across the BCI therapy intervention duration. Across sessions, BCI performance improves while VAS pain rating decreases. The shaded region depicts the median absolute deviation (MAD). (**d**) The median baseline θ power over the course of BCI therapy at electrode location F3 across participants with either significant pain reduction or consistent BCI performance over the ROC calculated optimal threshold (n = 4). Topography plots show median θ power across all electrodes during the first week (upper) and fourth week (lower, where baseline F3 θ was highest). Data were z-scored to baseline θ power, with shaded regions representing MAD.

At the group level, for the primary endpoint, there was a significant decrease in PSS and PIS (N = 6, Wilcoxon signed-rank, median decrease 1.29 ± 0.25 MAD, p = 0.03, q = 0.05 and 1.79 ± 1.10 MAD p = 0.03, q = 0.05, respectively, Fig. 4a and b, Supp. Table 4) after BCI intervention. For the secondary endpoint, the decrease in NPSI total score was not statistically significant (N = 6, Wilcoxon signed-rank, median decrease 5.25 ± 5 MAD p = 0.09, q = 0.09). Changes in NPSI and BPI throughout BCI intervention for each patient are visualized in Supp. Fig. 4. These results indicate improvements in pain severity and the extent to which pain interfered with day-to-day functions after participation in the BCI intervention. Changes in primary and secondary outcome (BPI, NPSI) for each patient before and after BCI intervention is shown in Supp. Table 4.

There were observed differences in BCI performance trends between patients, and thus, it was expected that changes in pain ratings would be different between patients. It is therefore important to acknowledge the individual changes in pain ratings over time and the overall relationship between these trends and frontal θ modulation. Median pain ratings decreased as median BCI performance accuracy increased across all patients over the BCI intervention (Fig. 4c). The overall trends in BCI accuracy, BCI bit rate, and VAS pain ratings for each patient are shown in Supp. Fig. 3. VAS was used to sample pain ratings during independent BCI training sessions, providing a transient metric of pain that we relate to electrophysiology and BCI performance. This enabled additional analysis to be conducted to relate electrophysiology, BCI performance metrics, and transient measures of pain. On an individual level, three patients showed a significant decrease in pain over time (n = 18, 15, 27, Spearman’s ρ = − 0.60, − 0.75, − 0.74; p = 0.01, 4.43e^−3^, 1.37e^−4^, q = 0.02, 0.01, 8.20e^−4^). Three of the patients significantly increased BCI performance over the intervention period (n = 18, 15, 27, Spearman’s ρ = 0.49, 0.58, 0.43; p = 0.04, 0.03, 0.03, q = 0.07, 0.07, 0.07), and one patient began the intervention with a near-perfect performance which decreased mildly over time. These four patients all showed a decrease in pain ratings before and after treatment (Supp. Fig. 3). Notably, while four of the six participants were classified as either performers (i.e. patients who had a significant increase in BCI performance over time) or VAS pain responders (i.e. patients who had a significant decrease in VAS ratings over time), all six patients had an improvement in primary outcome measures. Taken together, these results suggest a relationship between BCI performance, theta modulation, and pain ratings.

### Changes in power spectral density across BCI therapy runs

The study was designed to assess whether 6 weeks of BCI training led to significant resting-state frequency-band power changes. Patients classified as VAS pain responders and performers were those with significant pain decrease over time or with a significant increase in BCI performance over time, respectively (Supp. Fig. 3). For these patients, resting θ power at F3 increased over the course of therapy (Fig. 4d). Week four has the greatest median resting θ power across VAS pain responders and performers, which was notably the same week with the greatest BCI performance across all patients (Fig. 4c and d). There was no significant change in the resting power of any frequency band when comparing measures across BCI training sessions for all patients. Topography plots showing qualitative changes in resting power across all electrodes are shown in Supplementary Fig. 5.

### Specificity of theta modulation during BCI training

**Figure 5:**
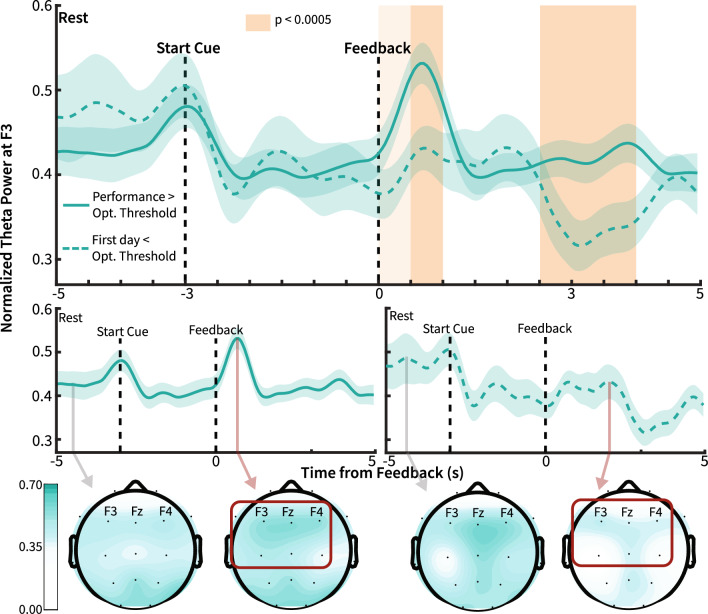
Differential θ modulation before and after BCI control. Top and Center: Patient normalized θ power during sessions with performance above optimal threshold (solid line) and first-day sessions below optimal performance threshold (dashed line). The optimal threshold was determined using ROC analysis to estimate the performance cutoff for BCI sessions with the greatest likelihood of significant θ modulation during neurofeedback. Black dashed lines indicate the onset of the start cue and neurofeedback spans. Orange shading indicates p values at durations without standard error overlap. Light orange shows duration without overlap between traces (500 ms window, n = 60, p = 0.006). Dark orange indicates durations of statistically significant differences between time traces (500 ms window, n = 60, Wilcoxon rank sum test, p < 0.0005, Bonferroni Corrected). The line shows averaged power values across each within-subject average, with the shaded region showing average standard error. Bottom: Topography of the normalized θ power during baseline and peak normalized θ during neurofeedback before and after BCI control. Theta power increases are localized to frontal channels during high BCI performance.

In order to identify the specificity of θ modulation and investigate whether frequency-specific modulation was different between high BCI control and the absence of BCI control, we compared the progression of frequency-specific power during BCI training trials. Figure 5 shows the median progression of normalized θ power across BCI training trials. The normalized θ power, averaged across all BCI sessions, was compared between high BCI performance and the absence of BCI control. These results exemplify the qualitative difference in θ power modulation specificity between sessions where patients did not yet acquire BCI control with sessions where subjects achieved BCI control. There were significant differences between the two power traces only during neurofeedback for F3 θ power, specifically 0.5 s to 1 s and 2.5 s to 4 s relative to neurofeedback onset. For α frequency band at F3, there was a significant difference in power from − 4 to − 3 s and 3 to 3.5 s. For β frequency band at F3, there was a significant difference from 2.5 to 3 s (for θ, α, and β, 500 ms window, n = 60, Wilcoxon rank sum test, p < 0.0005, Bonferroni corrected). There were no significant differences between power traces for δ and γ frequency bands. Traces for all power bands, including topography plots at each time point, are provided as videos in supplementary materials (Supp. Movie 1, Supp. Movie 2, Supp. Movie 3, Supp. Movie 4, Supp. Movie 5). Qualitatively, θ modulation was specific to the frontal region during high BCI performance, indicating the specificity of neuromodulation. Notably, there was a consistent decrease in α power during the neurofeedback period, irrespective of performance, which may indicate patient engagement in mental tasks (Supp. Movie 3)^42^.

### BCI performance as a proxy for theta modulation

**Figure 6:**
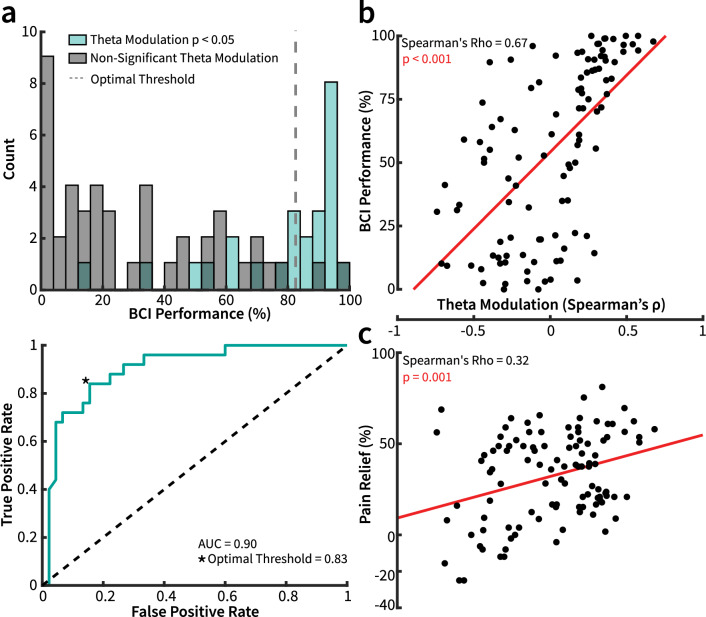
The relationship between θ modulation, BCI performance, and pain relief. (**a**) Top: Distribution of all BCI sessions binned by performance. Blue shaded bars represent sessions where participants significantly increased their θ power during the neurofeedback period relative to the pre-intervention baseline (Rank-Biserial Spearman’s Correlation, cyan: p < 0.05 and ρ > 0). The dashed line shows the optimal threshold calculated via ROC analysis. Bottom: ROC curve of the patient performance distribution. AUC of 0.90 and threshold of 0.83 or 83% for optimally classifying sessions with significant θ modulation. (**b**,**c**) θ modulation correlates with BCI performance as well as pain relief. (**b**) Relationship between BCI performance and θ modulation (n = 106, Spearman’s ρ = 0.67, p < 0.001) across all subjects and sessions. (**c**) Relationship between pain relief (percent pre-intervention baseline) and θ modulation (n = 106, Spearman’s ρ = 0.32, p = 0.001, Bonferroni Corrected alpha = 0.005) across all subjects and sessions.

To confirm that BCI performance was a proxy for θ modulation, and thus further validating the feasibility of this intervention, the distribution of BCI training sessions was plotted across all patients by performance with and without a significant increase of each frequency band at F3 during neurofeedback. Receiver operating characteristic (ROC) analysis revealed an area under the curve (AUC) of 0.73 for δ, 0.90 for θ, 0.84 for α, 0.76 for β, and 0.78 for γ (Fig. 6a, Supp. Fig. 6). BCI performance was the greatest predictor of power increase for θ frequency band at the BCI classification electrode F3. Also of note, the distributions of training session performance tended to congregate at either lower or higher performance values, with higher BCI performance corresponding to significant θ power increase during neurofeedback. This trend was not observed for any other frequency band. Sessions with significant F3 θ increase during neurofeedback also accounted for the greatest number of sessions with significant power increases across all frequency bands. There were 9 sessions with a significant δ increase, 38 with a significant θ increase, 23 with a significant α increase, 9 with a significant β increase, and 17 with a significant γ increase (Supp. Fig. 6). We found that θ modulation strength was positively correlated with BCI performance (n = 106, Spearman’s ρ = 0.67, p < 0.001; Fig. 6b).

### Correlation between pain reduction and theta power change at F3

After confirmation that the BCI intervention was able to specifically serve as a proxy for θ modulation, the relationship between pain relief and θ modulation was then examined. There was a direct positive correlation between pain relief and θ modulation increase during BCI training (n = 106, Spearman’s ρ = 0.32, p = 0.001, Bonferroni Corrected alpha = 0.005, Fig. 6c). This relationship was not significant for any other power band (Supp. Fig. 6). We tested this correlation for both pain ratings acquired before and after each BCI training session (n = 106, Spearman’s ρ = 0.29 and 0.32, p = 0.0009 and 0.001, respectively, Bonferroni Corrected alpha = 0.005). We found a significant correlation between pain relief and F3 θ modulation strength in both cases, which alludes to both direct pain relief as a function of each day’s performance, as well as an overall pain reduction as a function of increasing BCI performance throughout the duration of the therapy.

## Discussion

In this 6-week pilot study of patients with chronic upper extremity pain, a vibrotactile BCI therapy was used to increase frontal theta power and measure patient-reported changes in pain symptoms. Feasibility goals of patient retention and treatment protocol adherence were met. The results further indicate that this intervention reliably modulated frontal theta power and demonstrated feasibility in reducing pain. Specifically, this study met its proposed endpoint of a significant decrease in both pain interference score and pain severity score after BCI intervention in this group of patients. This result may allude to the generalizability of this treatment, as this patient group had heterogeneous demographics, initial symptoms, and trends in VAS pain ratings; however, future placebo-controlled clinical studies will be required to conclude the efficacy and limitations of this intervention. Throughout the vibrotactile BCI therapy, the magnitude of frontal θ modulation was significantly and positively correlated with pain relief as measured by VAS across BCI sessions. This was unique to frontal θ rhythms (F3 location) and was not present in any other frequency-specific power bands, indicating the specificity of θ modulation in pain relief. These results support our initial hypothesis that a non-invasive vibrotactile BCI device can feasibly target and reinforce brain activity associated with pain relief. These results warrant future work to validate this approach clinically and to better elucidate the mechanism that underlies how cortico-sensory coupling impacts chronic pain patient populations.

The experience of pain and pain relief has been shown to involve a complex neuronal network, often referred to as the pain neuromatrix. This model suggests contributions of bottom-up and top-down neural regulation of pain experience, including features beyond pain sensation such as emotional and salient components^26,43^. In neuromodulation studies using transcranial magnetic stimulation (TMS) or transcranial direct-current stimulation (tDCS), regions associated with top-down modulatory mechanisms of pain have been targeted, yielding successful reductions in pain symptoms^44^. TMS, applied to the dorsolateral prefrontal cortex in multiple experimental and chronic pain studies, led to pain reduction^45–51^. Similarly, various studies using tDCS of the prefrontal cortex have previously been shown to reduce pain. It is hypothesized that these techniques target the activity of pain perception and modulation areas, including the cingulate cortex, insula, amygdala, and thalamus^44^. These regions have previously been shown to play a major role in the top-down modulation of pain and are perturbed during chronic pain^7,52–55^. More recently, the electrophysiology of pain relief in acute and chronic settings has demonstrated that EEG frontal θ power increases are associated with the remittance of pain^27,28^. Source localization of these brain signals converges with previously identified anatomic sites from TMS, tDCS, and functional imaging studies^27,28,44,46–51,56–58^. These data motivated the electrode (F3) and frequency band θ configuration of this BCI system. The positive pilot results of this study support that the reinforcement of θ activity in this region may enhance the top-down capacity of critical sites such as the dorsolateral prefrontal and cingulate cortex to dampen noxious and aversive sensory perceptions.

Using a BCI to enhance the cortical physiology of *pain relief* as a therapeutic strategy is unique from prior approaches. Most studies using non-invasive neurofeedback for chronic pain have targeted pathological profiles of the sensory perception of pain, specifically by attempting to decrease activity associated with nociceptive processing^17,18,22^. A review by Patel et al. describes targeted neurofeedback systems, showing that the majority of existing work using neuromodulation for chronic pain has reinforced or inhibited EEG power at a variety of frequencies mainly over somatosensory areas (C3, Cr, Cz, T3, T4, P3, and P4 electrodes), yielding variable success in relieving symptoms of chronic pain^18^. The strategy for frequency band modulation was typically to increase α power (8–15 Hz), decrease β power (18–22 Hz), and occasionally decrease θ power. Only one study reinforced activity at frontal electrodes, but this was α at FP1^59^. These studies approached the problem of chronic pain by targeting pathological activity patterns associated with the encoding of pain over somatosensory areas. While a logical approach, the pathological profiles of pain are highly variable and can differ depending on the type of chronic pain^10,39,60^. Our novel approach targeted reinforcement of activity patterns associated with *pain relief* (i.e., left-frontal θ power increase), which was shown to be present in both relief from experimentally induced tonic pain and relief from chronic pain^27,28^. Given the substantial similarities of pain relief cortical physiology between these two studies with very different pain experiences, this physiology may be less variable across patients experiencing pain and enable a more broadly applicable BCI-mediated neurofeedback strategy.

Beyond the choice of cortical location and frequency band, the type and character of feedback are also critical. Prior BCI systems applied to chronic pain have used visual feedback that changed with brain signals associated with somatomotor intentions. For a system to be effective in neurorehabilitation of pain circuits, visual feedback is not likely to be optimal. To optimally induce remodeling via Hebbian learning, it is important to temporally couple a desired cortical physiology with *functionally relevant* sensory feedback to the affected anatomic location^12,13^. For example, in the setting of chronic stroke, it was critical to couple brain signals associated with motor intentions with proprioceptive kinematic feedback provided by a robotic exoskeleton to achieve a functional improvement^14,15,61^. In the setting of chronic pain, we aimed to couple cortical signals associated with pain relief (e.g., increased frontal θ power)^27,28^ with pain alleviating vibrotactile sensory stimulation in the distribution of pain^25,62,63^, to enable neural remodeling to occur. To this end, in addition to providing visual cues of the patient’s performance in controlling their frontal θ power, patients also received sensory-relevant vibrotactile feedback with stimulus intensity proportional to the magnitude of θ modulation. Previous evidence has shown that tactile feedback provides sensory-relevant feedback and can improve BCI control^64,65^. Similarly, the use of vibrotactile feedback, which has previously been shown to lead to transient pain relief^62,63,66^, can act as a potent reinforcer to the central physiology of pain relief. From a Hebbian standpoint, the use of a BCI is especially salient because transient pain relief co-occurs precisely in time with the θ changes in the frontal lobe. While an important consideration for pain, this general configuration of BCI-mediated cortical-sensory coupling has yet to be optimized. It is unclear what are the optimal vibrotactile parameters in terms of intensity, timescale, and vibration frequency to best support central neural remodeling to lead to long-term pain relief.

While the central results of this pilot study are clinical improvements with the use of the system, there are also intriguing neurophysiology findings that support the notion of neural remodeling occurring. Specifically, baseline θ power (in the absence of task) changed over time, primarily in the left hemisphere. This change occurred both in frontal and central regions (EEG electrodes F3, C3, P3). There is prior evidence that resting-state dynamics of θ rhythms have been considered a biomarker of BCI-mediated changes in the brain^67^. In the setting of BCI-induced motor rehabilitation in chronic stroke, the degree of change in resting-state θ rhythms was closely correlated with the degree of motor improvement. Similarly, those patients with significant improvement in their pain demonstrated notable changes in their fronto-central baseline power. While too early to make definitive conclusions, these θ power changes merit future exploration as a potential EEG biomarker for assessing the impact of BCI-mediated pain therapy.

While the results demonstrate the potential of a novel BCI-mediated neuromodulation therapy for chronic pain, there are several limitations that merit consideration. Primarily, this clinical pilot included only six patients making more generalized claims limited. This study also lacked a control group to differentiate between the effect of θ-driven vibrotactile stimulation and placebo effects, particularly considering the setting of in-person visits to our research facility. In the future, it will be important to increase the number of patients to power the study adequately and include an additional pseudo-neurofeedback control group which will receive the visual and vibrotactile feedback, in comparable dosages, without any correlation to brain activity modulation. This will better define the role of frontal θ modulation in reducing pain, and whether coupling the vibrotactile and visual neurofeedback is effective in mitigating pain symptoms. Further, this control condition will be able to decouple the effect of frontal θ reinforcement and vibrotactile feedback over a longitudinal intervention period on observed therapeutic outcomes. A larger study will also be necessary to define electrophysiological biomarkers such as general changes in power, connectivity, and phase-amplitude coupling, which have previously been shown to be associated with pathology in chronic pain. The dosage (i.e., how long patients should be treated) and the durability of the treatment (i.e., how long the effect lasts) will need to be defined in future studies. Future iterations of this BCI will also involve the refinement of parameters, including feature discrimination and feedback optimization, to maximize therapeutic effect. As an initial pilot study, our approach to feature selection and feedback parameterization was simple. In the future, machine learning approaches can be used in several ways to increase BCI-literacy and therapeutic output^68^. Variance in spectral properties between patient EEG implies feature weights can be modified in order to best tailor BCIs to individual patients. Specifically, classification training can be used to select the best spectral targets for reinforcement in order to maximize therapeutic benefit. Given the importance of feedback in BCIs, machine learning methods can also be used to converge on vibrotactile stimulation feedback parameters to maximize reinforcement of frontal θ power and therapeutic outcome.

This pilot study aimed to assess the feasibility of a novel frontal θ driven BCI mediated vibrotactile therapy for chronic pain. We demonstrated the effectiveness of frontal θ reinforcement using our BCI, and that reductions in patient pain severity and pain interference scores can be achieved through use of our system.

## Methods

### Study participants

The study was approved by the IRB of Washington University School of Medicine, and all experimental procedures were conformed to the standards set by the latest revision of the Declaration of Helsinki. All participants signed an informed consent form prior to participation. Inclusion criteria were (1) age ≥ 18; (2) upper extremity pain located distally to the elbow (e.g. hand, wrist, forearm); (3) continuation of painful symptoms despite at least 2 previous different interventions, including topical or oral NSAID treatment, local corticosteroid injection, or surgery; (4) pain duration ≥ 4 months; (5) daily moderate to severe pain with an average intensity of ≥ 4 on 0–10 Numerical Rating Scale (NRS); (6) ability to attend training sessions for 3–5 h a week. Exclusion criteria were (1) inability or unwillingness to provide informed consent; (2) significant psychiatric or neurological condition (e.g., dementia, epilepsy, stroke) known to cause changes in EEG; (3) inability to wear a head cap due to non-healing wounds or other scalp disorders; (4) Inability to have vibrating elements put on the skin due to skin ulceration, non-healing wounds, or other skin conditions; (5) Complete lack of sensation in the testable limb; (6) Pain located proximally to the elbow; (7) The use of caffeinated products or nicotine three hours prior to each study session.

### Feasibility assessment

For the scope of this pilot study, intervention feasibility was assessed on various criteria. Patient adherence and retention were reflected in participation and treatment frequency. Patients were instructed to attend BCI training sessions for a minimum of 3 days a week over the intervention duration. Patients were encouraged to participate in each session for 30–45 min but were accommodated for scheduling conflicts. During treatment sessions, patients were instructed to allocate as much attention as possible to the BCI training task (main intervention), to turn off communication devices, and to talk only during the allotted break periods. Those unable to adhere to this regimen during the intervention were given the option of restarting the treatment (i.e., restarting the protocol from the first week). The inability to adhere to the treatment schedule resulted in withdrawal from the study. Data from patients who did not complete 5 weeks of training were not analyzed. Successful patient retention was defined as completing at least 5 weeks of intervention for at least 3 days a week. Weekly session duration was assessed to determine patient adherence and retention. Intervention safety, risk, and comfort were determined by patient-reported feedback after each BCI training session. BCI patients were instructed to verbalize any discomfort they experienced and whether they felt any part of the intervention was unreasonable.

The success of this pilot’s objectives and hypotheses are predicated on the intervention’s design in successfully inducing modulation in patient θ power during BCI training. Two main steps were used to assess the feasibility of the intervention in producing and detecting modulation in left frontal θ. First, a pre-screening frontal θ modulation task was implemented. This task aimed to assess each participant’s ability to modulate θ activity and whether this modulation could be detected via analysis. Second, the qualitative changes in θ power during consecutive days of BCI training were assessed and compared to BCI performance scores and pain reduction. To this end, we evaluated whether BCI performance could serve as a proxy for θ modulation strength and whether the degree of θ modulation was related to changes in pain ratings.

### Baseline metrics

Patients completed self-report questionnaires, underwent cognitive testing, baseline brain activity recordings, and screening for task-specific θ modulation prior to any BCI-intervention. Specifically, participants rated their pain intensity on VAS, completed BPI, NPSI, and the PCS. In addition, HADS was used to determine patient anxiety and depressive symptoms. QST was performed to assess patients’ somatosensory profile^69–71^. The EEG recordings took place in a quiet study room with minimal outside disturbance. Participants were comfortably seated in a stationary chair and were asked to limit their movements during brain activity recordings. The details of the methods used to obtain these metrics are described in the Supplemental Methods.

### System overview

The BCI system consisted of a DSI-24 EEG recording system (Wearable Sensing, San Diego, CA, USA) connected to a Windows PC Laptop via Bluetooth, enabling easy and comfortable measurement of high-fidelity EEG signals in a laboratory environment, with minimal setup time. The HVSA provided real-time vibrotactile neurofeedback, and a monitor provided visual feedback in the form of a vertical cursor task. BCI2000, a general-purpose, open-source modular software, was used to manage data acquisition, real-time signal processing, application execution, event logging, and system synchronization^72^. This device consisted of a custom-designed adjustable frame able to fit most hand sizes, with two (upper and lower) grids of 24 small vibrating motors contacting both sides of the hand. This device executed vibration commands via an Arduino Microcontroller connected to the PC.

### Real-time signal processing

A real-time spatial and spectral filter was applied to acquired EEG data to extract frontal-midline θ power. A 1 Hz high pass and 50 Hz low pass filter were applied in real-time to avoid the influence of artifacts from line noise or channel drift on BCI classification accuracy. Signals were common average re-referenced, then spatially filtered such that F3, Fz, and F4 electrodes were selected as feature input channels. θ power was calculated in real-time by an autoregressive (AR) spectral estimation using a 500 ms sliding window. The AR estimation was selected as it provides a computationally efficient means for real-time spectral estimation and has been shown to have high spectral resolution even for shorter data segments^73^. 4–6 Hz power was defined as the as the driving spectral feature. 4–6 Hz was chosen over 4–7 Hz to minimize the effect of α band variance on the spectral filter. Previous evidence has suggested task-specific and resting-state variance of α power both within and between populations of subjects^74^. Further, the proximity of commonly defined α frequencies to our desired spectral feature of θ could have potentially influenced the specificity of our spectral estimations due to spectral leakage^75^. To avoid these confounds, we chose not to include the frequencies closest to either θ or α as part of our real-time feature extraction. Further elaboration on the selection of 4–6 Hz can be found in Supplemental Results. This real-time processing method enabled computationally efficient processing in order to minimize the latency between real-time processing of electrophysiology and execution of neurofeedback commands.

### Classifier output calibration and normalization

To define baseline θ power for real-time classification during BCI training for each patient, a zero-mean distribution of the subject’s resting θ power at the F3 channel was generated. This was done during a set of ten “adaptation” BCI trials, where the BCI2000 adaptation function was enabled. This function optimized normalization values for calculating relative changes in θ power by determining a zero-mean value for θ power during rest, and an arbitrary gain value which aided in magnifying output commands. This gain value was also optimized such that patient eyeblink artifacts, which lead to transient increases in δ-θ power, did not significantly influence BCI performance metrics (primarily by regulating the rate of trial completion, such that consecutive eyeblinks alone could not lead to trial success). After ten of these adaptation trials, a set zero mean value was uniquely used for the remainder of the BCI therapy for each patient. The output of the classifier was thus a θ power value normalized to the baseline zero-mean value, multiplied by the determined gain value:

$$output=\left(input-ZeroMeanValue\right)*AdaptiveGainValue$$

The output value was used to drive neurofeedback, with positive values indicating increases in θ power at F3 and negative values indicating decreases. The overall magnitude of the output value was reflected in the velocity of the visual cursor and the intensity of the vibrotactile feedback, as described below.

### BCI training intervention

Patients underwent at least three one-hour BCI training sessions per week for at least 5 weeks, but no more than 6 weeks (intervention duration). This duration and weekly session number were selected based on intervention durations of previous longitudinal BCI or neurofeedback therapies^18,76^. VAS pain scores were obtained from patients upon arrival to the study room and immediately after each day’s BCI training session. Baseline recordings were obtained via EEG during the pre-BCI training period each day (5 min), during the BCI training session (up to 45 min), and during each day’s post-BCI training session (5 min). The study design is shown in Fig. 2a. During baseline recordings (pre and post BCI training), patients were asked not to perform specific mental tasks and to remain calm and relaxed while remaining awake. A fixation cross was presented on the display, and patients were asked to fix their gaze on the cross. Patients were instructed to blink at a natural rate, ensuring that they did not blink inconsistently between parts of the BCI trials. After the pre-BCI training recording, patients were asked to place their affected hand into the HVSA.

BCI training consisted of three 15-min blocks. Each trial consisted of a ten-second baseline period, a three-second start cue period, a 20-s limit neurofeedback period, a three-second stop cue, and a seven-second post stop-cue pre-baseline buffer period (Fig. 2b). The subjects were presented with a yellow on-screen cursor which remained stationary at the center of the screen during non-neurofeedback periods. At the top of the screen, a green rectangle indicated the target and remained on top of the screen during all periods. The green rectangle served as the target and would always appear at the top of the screen in the same location. During the start cue, the word “Start” was presented in the center of the cursor, and a random prime number between 3 and 19 appeared in the center of the top rectangle in cyan. This prime number remained in the rectangle for the duration of the neurofeedback period, as an option for patients to use serial subtraction as the driving task for frontal θ modulation if needed. Additional methods regarding the role of the prime numbers are provided in Supplemental Methods. After three seconds, the word “Start” would disappear, and the neurofeedback period would begin. During the neurofeedback period, the center cursor would either move up if the subject increased their left frontal-midline θ power above baseline or move down if their left frontal-midline θ power decreased below baseline average. The speed of cursor movement corresponded to the magnitude of increase or decrease (see description of intervention). During neurofeedback, the HVSA would stimulate the participant’s affected area when there was an increase in θ power. The intensity of the vibration corresponded to the magnitude of θ power increase. The vibration was pulsed at a random frequency selected from 4, 5, 6, or 7 Hz. If the participant was able to reach the target by moving the cursor to the top rectangle within the allocated 20-s period, the stop cue would initiate, and a burst of vibration at maximum intensity was delivered for the duration of the three-second stop period. If the participant could not reach the target within the allocated time limit, the stop cue would initiate with no vibration delivered. The stop cue consisted of the cursor returning to the center of the screen, and the word “Stop” was displayed in the center of the cursor. The seven-second buffer period allowed for any brain activity changes resulting from the stop period and the neurofeedback period to wash out such that the activity recorded during the baseline period was unaffected. Figure 2b shows the design of a single trial within a training session. Additional information describing the methodology and characteristics of the continuous BCI used in this study is shown in Supp. Table 5. At the end of each week on Friday, patients received an email containing a survey. The survey was used to obtain self-reported BPI and NPSI metrics each week outside of the experimental environment.

### Data collection and outcome measures

Electrophysiological activity recordings throughout the therapy duration were carried out using dry-surface EEG. Primary and secondary outcome measures were self-reported by patients.

### EEG recording

EEG was recorded using a 24 wireless dry electrode headset in an international 10–20 system (DSI 24, Wearable Sensing, San Diego, CA, USA). EEG was referenced to the Pz electrode and sampled at 300 Hz with a ground electrode placed on the earlobe. Electrode impedance was maintained below 10 kΩ.

### Primary and secondary outcome measures

The primary outcome measure was the BPI PIS sub-score. Secondary outcome measures were the NPSI total score (50 points total, consisting of sub-scores of 10 points each for burning pain, pressing pain, paroxysmal pain, evoked pain, and paraesthesia/dysaesthesia, where higher scores reflect more severe symptoms of a specific quality) and BPI PSS sub-score, which were recorded at the end of each week for each patient^77,78^. These outcomes, especially BPI, are considered robust and reliable outcome measures for trials in patients with chronic pain, where higher scores indicate worse or more severe symptoms related to the quality and intensity of pain (PSS, 0–10 with 0 = no pain and 10 = worst imaginable pain), and the degree that the pain symptoms interfere with everyday life (PIS, 0–10 with 0 = no interference and 10 = interferes completely)^79,80^. These metrics have previously been shown to have high test–retest reliability and have been adapted to a variety of diseases and types of pain^77,81–83^. For reference, previous work has reported that 1-point changes in PIS reflect a typical 0.5 standard deviation change, and is a benchmark for minimal clinical importance, and improvements of 2 points or greater reflect moderate or meaningful changes. For pain severity, recorded as PSS, 10% point changes were considered minimally important, while reductions of 30% or more were considered to be clinically important^83^. These metrics were acquired via patient response on a survey emailed to each patient at the end of each week. Survey outreach was handled by the REDCap database/survey management software. VAS scores (additional secondary outcome, where 0 = ‘no pain’ and 100 = ‘worst pain imaginable’) were acquired from patients before and after each BCI session and used for secondary analysis to relate changes in pain ratings, BCI performance, and electrophysiological features^84^.

### Offline data analysis

Processing of electrophysiological data and statistical analysis was conducted in MATLAB environment (Mathworks, Nattick, MA, USA).

### EEG processing

EEG data were preprocessed by first applying a 1 Hz high pass and 40 Hz low pass Butterworth filter to the signal. Next, high amplitude signal artifacts were removed by thresholding above each subject’s manually determined eyeblink artifact threshold. This was done for each data file qualitatively by manually identifying the largest magnitude eyeblink artifact and then setting the threshold above this value. The threshold was set above the magnitude of the eyeblink artifact because independent component analysis (ICA) was used to remove eyeblink artifacts, which minimized the amount of electrophysiological activity removed from the data. Signals containing samples above this threshold were deleted and then smoothed using a five-point moving average interpolation. The signal was then common average re-referenced, and ICA was performed to remove eyeblinks, eye saccades, lateral eye movements, muscle-related, and cardiac-related artifacts from the data^85^.

Band-limited amplitude time series was extracted by applying a Butterworth with frequency cutoffs as follows: 1–3 Hz for delta (δ), 4–7 Hz for θ, 7–13 Hz for alpha (α), 13–30 Hz for beta (β), and 30–40 Hz for gamma (γ). Power envelopes for each signal were calculated by squaring the absolute value of the Hilbert transform of the time series. The mean power envelope within each relevant timeframe was used for overall power calculations.

For electrophysiological comparisons within and between subjects across multiple days, power values were normalized to baseline recordings by z-scoring all power envelope time series to power envelope from EEG data recorded before any BCI intervention.

For analysis of continuous, relative power changes within single BCI sessions, a time–frequency analysis was conducted by time locking pre-processed signals acquired from each session across each subject to trial start and neurofeedback onset. Time–frequency was determined by the mean Fast Fourier Transform of all trials with Hamming window tapering^85^. Time–frequency signals were binned into power bands as described above, then normalized to the minimum and maximum power values within each session, exemplifying the spatial–temporal modulation of different frequency-specific activities within each BCI training session.

For analysis of spectral properties between conditions, specifically, to visualize qualitative differences in power spectral density plots, Welch’s power spectral density estimate was used with a Hamming window equal to ten percent of the epoch duration^86^. This analysis was applied to demonstrate qualitative differences in θ power modulation during the pre-screening mental arithmetic task and BCI training.

### Statistical analysis

Statistical tests were conducted using non-parametric methods. None of the implemented statistics were used for concluding clinical or mechanistic outcomes. Rather, the tests employed for this study aimed at quantitatively supporting the feasibility and potential of the device for larger, placebo-controlled clinical trials and for identifying potentially relevant mechanisms evoked by BCI intervention that justify further investigation. For correlation analysis conducted between pain intensity and treatment time, BCI performance (measured as BCI accuracy: # trials goals reached/# total trials) and treatment time, BCI bit rate and treatment time, θ power modulation and BCI performance, and θ power modulation and pain relief, Spearman’s correlation was used. Statistical power analysis showed that 87 sessions were ample to detect a significant correlation strength of 0.3 (Moderate Correlation) with a power of 95%. The present study includes data from 106 BCI sessions. BCI throughput (bit rate) was calculated using Fitts’s metrics in bits per second as an alternative means of measuring BCI performance in addition to accuracy^87^. Throughput (bits per second) was calculated as the cursor task’s index of difficulty which was equal to the log_2_(1 + 238) over the time to reach the target for each trial. 238 was the total distance the cursor traveled in pixels to reach the goal. For trials where the goal was not reached, the time to reach the target used for the calculation was 20 s, as this was the maximum duration participants had to reach the goal. Patients with a significant increase in BCI performance accuracy over time were classified as performers. Patients with significant decreases in pain over time were classified as VAS pain responders. To determine the significance and directionality of power modulation from baseline, we used a non-parametric rank-biserial Spearman’s correlation^88^. This method enabled correlation between continuous ranked variables (i.e., EEG data from two conditions) and a dichotomy (i.e., a generated data vector of − 1 s and 1 s corresponding to the lengths of either EEG data conditions). We chose this method over a Wilcoxon rank-sum test as it reflected the directionality and magnitude of significantly different frequency-specific power. To compare group-level changes in the outcome measures, a two-sided Wilcoxon signed-rank test was used. For statistical comparisons of power differences at single channels between conditions, a two-sided Wilcoxon rank-sum test was used. To account for multiple comparisons for these statistical tests, a Benjamini and Hochberg procedure was used to calculate the false discovery rate (q-value), as this test has shown to be more conservative and better fit for fewer comparisons^89^. Due to the small study size, the median was used as the central tendency value for comparisons across all subjects, and the median absolute deviation (MAD) was used for variance. The mean and standard error were used for comparisons of electrophysiological data consisting of multiple trials. For detecting whether there were significant differences between weekly intervention participation time, a Kruskal–Wallis test was used^90^. Repeated measurements were taken across all participants for both clinical and electrophysiological variables.

ROC analysis was performed to assess whether BCI performance was an accurate proxy for θ modulation. For each session, we evaluated whether significant θ modulation was achieved by performing a rank-biserial Spearman’s correlation between pre-BCI intervention baseline θ power and each session’s neurofeedback period θ power at F3. Baseline epochs and neurofeedback epochs were the same lengths. We defined sessions with successful θ power increases as those with a p < 0.05 and Spearman’s ρ > 0. We then performed ROC analysis on the distribution of all sessions, including sessions with or without significant increases in θ power at F3. An optimal performance threshold for identifying a BCI performance value most likely to reflect θ power increase at F3 was determined by maximizing the true positive probability (i.e., session performance truly reflects significant θ power increase) and minimizing false positive probability (i.e., session performance reflects significant θ increase without actual increase). Sessions with high BCI performance were defined as sessions with performance above the optimal threshold calculated with ROC analysis (83%). Sessions with no BCI control (naïve sessions) were defined as each patient’s first BCI training session with performance below the optimal threshold.

For comparisons of normalized band-power differences between traces generated by these groups, we compared data within 500 ms bins using a two-sided Wilcoxon rank-sum test. Permutation tests (1000 iterations) were performed for all correlations, a biserial correlation analysis. We next compared the relationship between magnitude θ increase (Spearman’s ρ) with BCI performance and pain relief using Spearman’s correlation. Significance levels were Bonferroni corrected for these tests.

### Supplementary Information

Supplementary Information.

Supplementary Video 1.

Supplementary Video 2.

Supplementary Video 3.

Supplementary Video 4.

Supplementary Video 5.

## Data Availability

The authors would like to indicate that our data is available upon reasonable request by contacting the corresponding author or the first author, Phillip Demarest (d.phillip@wustl.edu).
