# Supplementary material for: A novel theta-controlled vibrotactile brain–computer interface to treat chronic pain: a pilot study
Source: Sci Rep. 2024 Feb 10;14:3433. doi: 10.1038/s41598-024-53261-3 (PMC10858946; doi:10.1038/s41598-024-53261-3)
Supplement: Supplementary file 1 — Supplementary Information. [file 41598_2024_53261_MOESM1_ESM.docx]

A Novel Theta-Controlled Vibrotactile Brain-Computer Interface to Treat Chronic ~~P~~ain: A Pilot Study

Phillip Demarest^1,2^, Nabi Rustamov^1,3^, James Swift^1,3^, Tao Xie^1,3^, Markus Adamek^1,3^, Hohyun Cho^1,3^, Elizabeth Wilson^4,5^, Zhuangyu Han^1,2^, Alexander Belsten^1,3^, Nicholas Luczak^1,3^, Peter Brunner^1,2,3^, Simon Haroutounian^4,5^, *Eric C. Leuthardt^1,2,3^

**SUPPLEMENTAL METHODS**

**Evaluation of Hypersensitivity:** Mapping participant’s affected extremity for areas of spontaneous pain and thermal hypo/hypersensitivity was done by exposing the area to a 20°C cold stimulus, 40°C warm stimulus (Thermal Roller, Somedic, Sweden), brush (SENSELab Brush-05, Somedic, Sweden), and pinprick (Von Frey filament #6.10, applied pressure 980mN)^1^.

**Quantitative Sensory Testing** (QST) was performed at baseline using an abbreviated version of the German Research Network on Neuropathic Pain (DFNS) protocol to determine differential sensory profiles of each participant’s affected (most painful) and unaffected (non-painful) regions^2,3^. This was used to assess warm and cold detection (WDT and CDT) thresholds, heat and cold pain thresholds (HPT and CPT), mechanical detection and pain thresholds (MDT and MPT), presence of wind-up (enhanced temporal summation) to pinprick, vibration detection thresholds (VDT), and pressure pain threshold (PPT), and conditioned pain modulation (CPM). The Thermal Sensory Analyzer (TSA-II or PATHWAY platform; Medoc, Ramat Yishai, Israel) was used to determine WDT, CDT, HPT, and CP ^4,5^. A set of standardized Semmes-Weinstein monofilaments (0.25, 0.5, 1, 2, 4, 8, 16, 32, 64, 128, and 256mN; North Coast, USA) was used to assess MDT for each participant’s affected and unaffected area. A set of calibrated blunt metal probes with applied force 8-512mN (MRC systems, Germany) was used to determine the MPT. Wind-up ratio (WUR) was assessed using a #6.10 von Frey filament (. 980mN) and calculated as the ratio between pain intensity elicited by a train of ten 1Hz stimuli versus a single stimulus value was calculated^6^. Each participant’s frequency of vibration detection threshold was determined using a clinical tuning fork (64Hz, 8/8 scale)^7,8^. A handheld algometer (Wagner Instruments) was used to determine each participant’s PPT following continuous pressure application at a 0.5kg/sec rate. The test sites used for affected and control sites were along the same muscle. A cold-water bath and the Thermal Sensory Analyzer were used to assess each participant’s CPM. CPM was evaluated by determining the change in elicited thermal pain rating before versus during exposure to a conditioning stimulus (cold water bath maintained at 12°C). The test stimulus was determined as the probe temperature, which elicited a pain intensity of 50 on a 0-100 NRS in each participant. First, the Pain-50 stimulus was applied, and the subject-reported pain intensity (NRS) evoked by the stimulus was documented. This procedure was repeated twice. Next, the Pain-50 stimulus was applied during the last 30 seconds of a 60-second contralateral cold conditioning, and the participant was asked to report the pain intensity elicited by the thermal probe. This was repeated twice. The difference between the reported pain intensity of the stimulus before and during conditioning was the CPM magnitude. A CPM <0 implies efficient descending pain modulation.

**Description of Pre-Screening Session (Task-Specific θ Modulation):** To train subjects to control a BCI using frontal-θ power, a task was provided to initiate modulation of this pattern. We collected EEG during a serial subtraction mental arithmetic task to assess each participant's characteristic θ modulation profile. It has been previously shown that mental arithmetic, calculations, and mental tasks increase frontal midline θ activity. We used this observation to pre-screen participants for task-specific modulation^9–11^. This strategy was used for all participants to induce initial θ modulation, which could be used to exert BCI control. Baseline EEG data were collected for five minutes before the experiment. The task design consisted of two 25-trial blocks. Before assessment recording, the subjects were given randomly generated three-digit multiple of 50. This number would be used for the serial subtraction task. Each trial consisted of a five-second task period where participants were asked to engage in serial subtraction and a five-second baseline period where subjects were asked to fix their gaze on a fixation cross presented at the center of the display without performing any mental tasks. During the task period, a pseudorandom prime number between 3 and 19 was selected and presented to the participant in the middle of the screen. The participants were asked to serially subtract that prime number from the three-digit multiple of 50 until that number disappeared and the baseline fixation cross was presented. Offline analysis was conducted to quantify the degree of θ modulation and quantify the spectral properties of their activity modulation.

**Specific instructions given to patients when engaging in BCI task:** Participants were informed about the context of the BCI intervention during the baseline visit, and instructions were reiterated briefly at the beginning of each BCI training session. Participants were informed that the research group had identified an EEG biomarker (frontal θ power) that may have therapeutic implications for chronic pain symptoms and that the present study wanted to evaluate whether BCI could be leveraged to target and reinforce this activity. Participants were asked to watch an informative video which provided instructions and context to what the BCI intervention would entail. They were informed that during BCI training, successful increases in frontal θ power would result in ascending cursor movement towards a goal on the screen, and concurrent vibrotactile stimulation of the hand on their affected limb. Patients were given examples of previous methods identified in the literature, where increases in frontal θ power have been observed. Patients were then told that serial subtraction would be the main task recommended by the research group to begin modulating frontal θ power. They were told that during BCI training, a series of prime numbers would appear on the goal, and they would have the option to use these prime numbers to guide a serial subtraction task. They were told that serial subtractions involved repeatedly subtracting the same number (in this context, the prime number that appeared on the screen) from a provided number (which was a randomly generated multiple of 50 between 1000 and 2000, and provided to the patient before each BCI session). While we recommended this method to the patients, the patients were also provided with alternative examples, including meditation and other cognitive control-related tasks. We encouraged the patients to try various methods and that they would always have the option to default to the serial subtraction tasks if needed. Participants were told that any kind of cognitive task leading to vibrotactile feedback and moving the cursor to the goal would be an appropriate means to increase frontal θ power.

**SUPPLEMENTAL RESULTS**

**Spectral Band Feature Selection Influences Variance of θ Power.** To minimize the effect of alpha power variance on our real-time spectral estimations of θ power, the driving BCI feature was selected to be 4-6 Hz. Initial considerations were made based on prior evidence demonstrating the divergent frequency ranges of alpha amplitude, where the characteristic frequency ranges have previously been reported to be anywhere between 6 Hz and 13 Hz^12,13^. Further, in order to mitigate the potential effect of blurring on the θ spectral estimation and the confounding impact of blurring from neighboring frequency bands (in this case alpha), we established a buffer area between the two frequency bands between 6 Hz and 8 Hz. For the purposes of this pilot study, we wanted to minimize the impact of inadvertent alpha modulation and subsequent neurofeedback on our goal of reinforcing frontal θ power. The potentially confounding effect of including spectral power within this range was tested by calculating the variance of θ power change during the task-specific θ modulation screening. The variance of θ power change was calculated across all trials and subjects using either 4-6 Hz, 4-7 Hz, or 4-8 Hz as the frequency range for θ power. The variance in θ power change was found to be 0.079 µV^2^/Hz when using 4-6 Hz, 0.082 µV^2^/Hz when using 4-7 Hz, and 0.108 µV^2^/Hz when using 4-8 Hz.


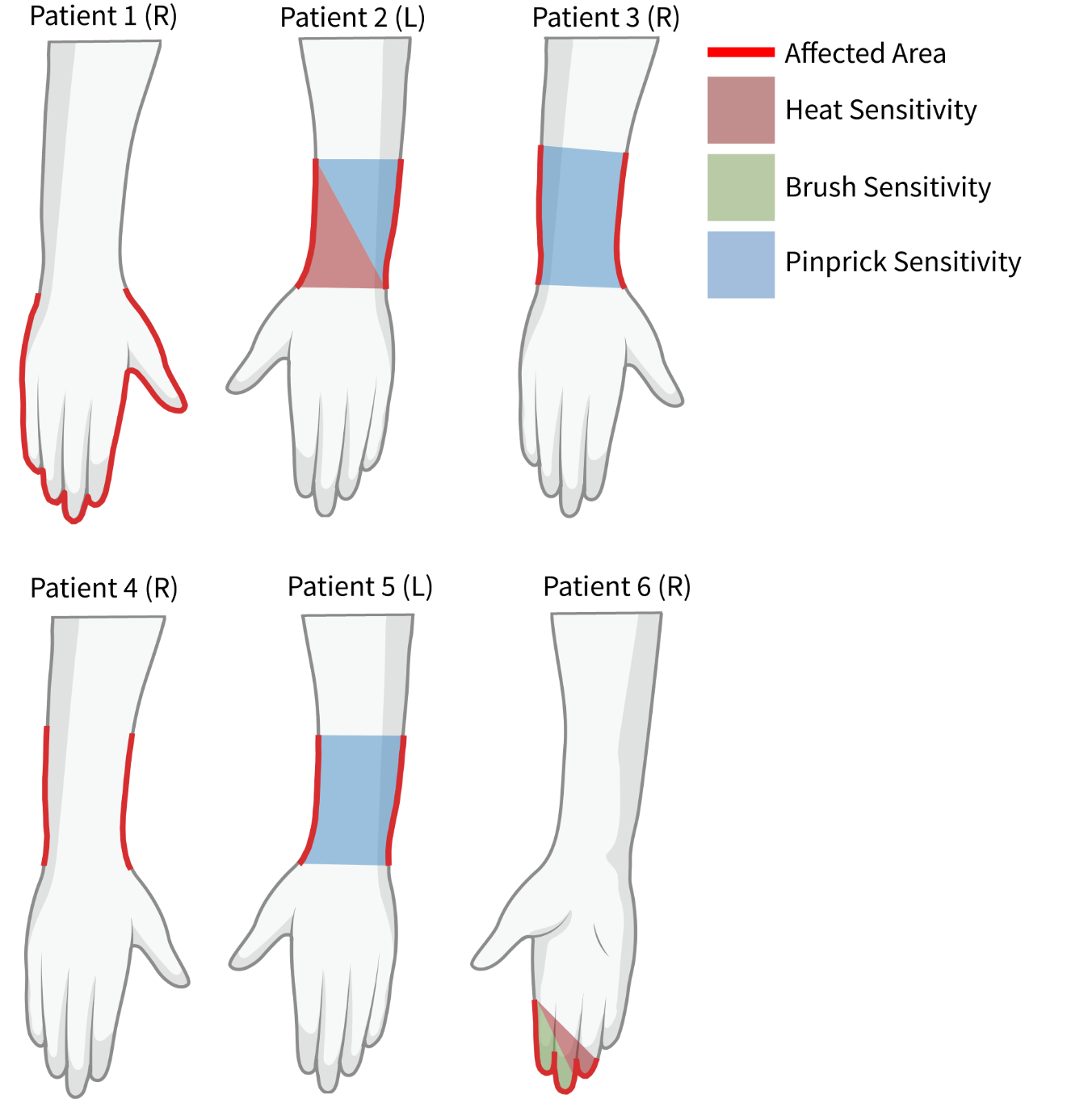


**Supplemental Figure 1. Schematic of handedness, location, and hypersensitivity type of each patient’s affected area.**

The affected region is shown in red. Heat sensitivity is shown in red shading. Brush sensitivity is shown in green shading. Pinprick sensitivity is shown in blue shading.


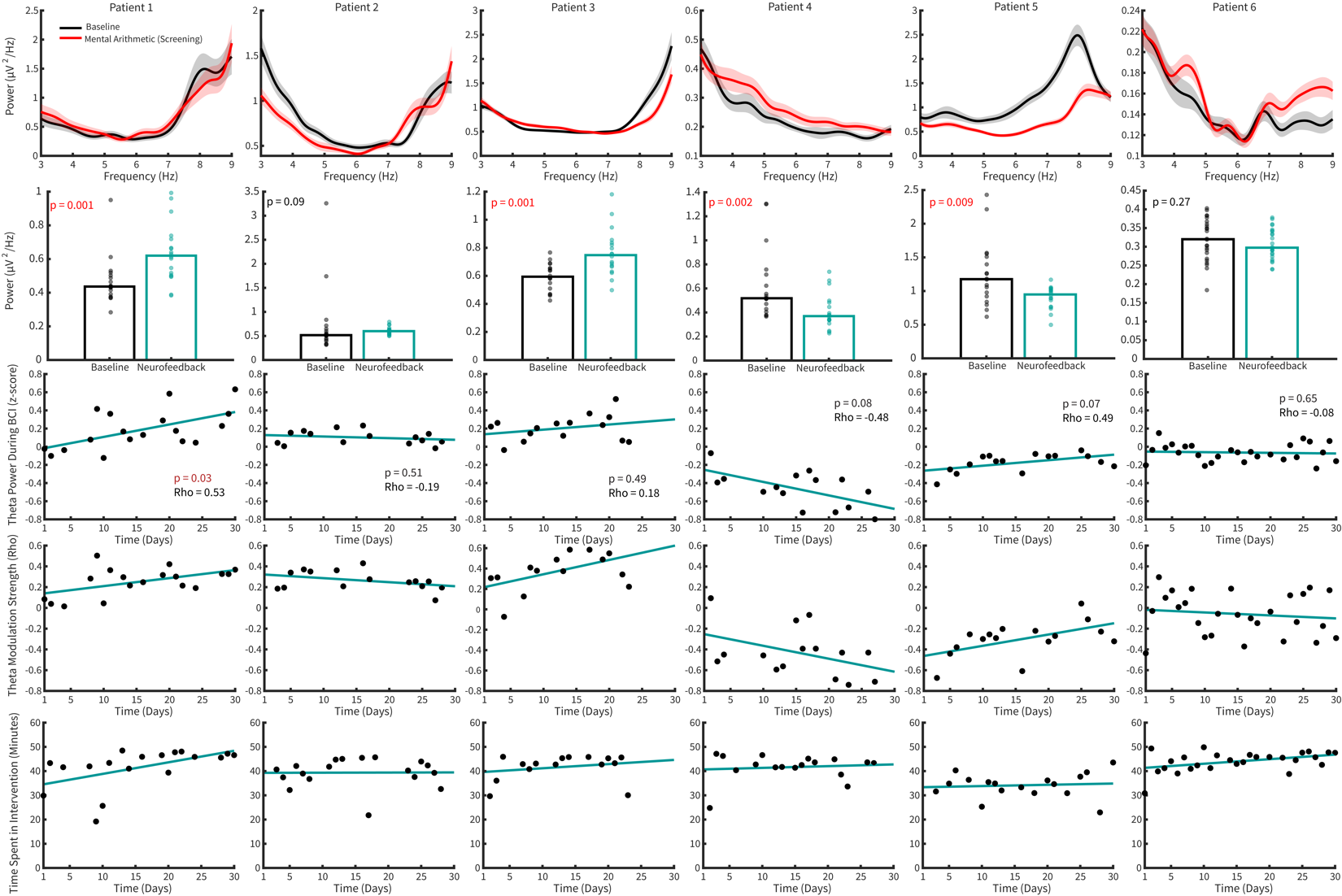


**Supplemental Figure 2. Visualization of θ modulation.**

(From top to bottom) Power spectral density plots showing differences in frontal θ modulation during a serial subtraction mental arithmetic task (shading represents standard error). Comparison of frontal θ power during pre-BCI intervention baseline (black), and during BCI-neurofeedback (Wilcoxon rank sum, alpha = 0.05). Normalized θ power across BCI sessions over time (Spearman’s Correlation). θ modulation strength over time across BCI sessions. Total session time for each BCI training day. The blue line shows the overall trend by a line of best fit.


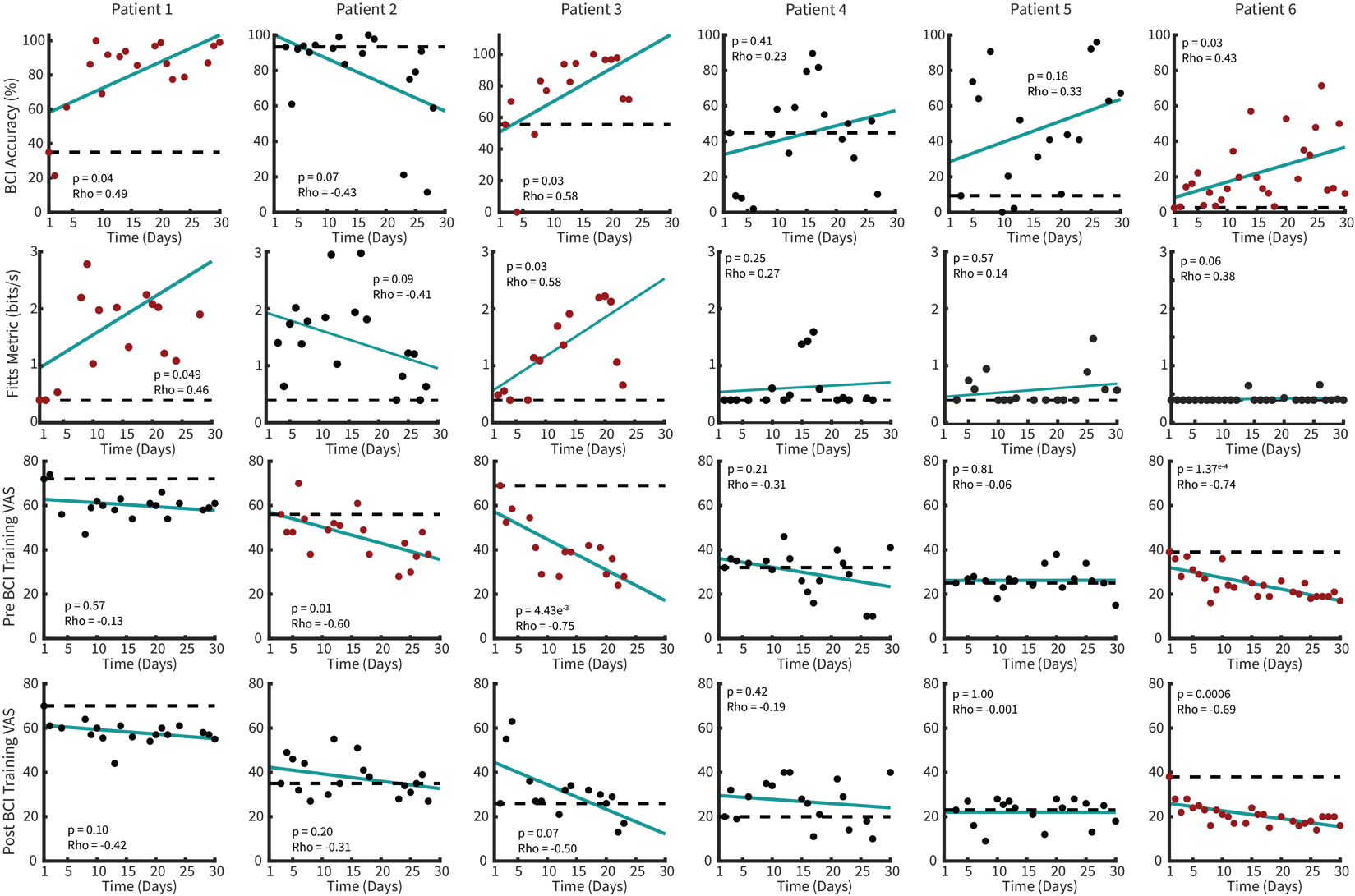


**Supplemental Figure 3. Individual patient BCI performance and pain over BCI intervention.**

(From top to bottom) Each BCI session accuracy (completed trials/total trials) where the dotted line is accuracy during the first BCI day, median Fitts Metric of bit rate (bits/s) where the dashed line is the bit rate calculated for maximum duration of BCI control, pre-session VAS where the dashed line is the first pre-session VAS score, and post-session VAS where the dashed line is the first post-session VAS score on each day for each patient. Significant changes over time are shown by red data points (Spearman correlation). The blue line shows the overall trend by a line of best fit.


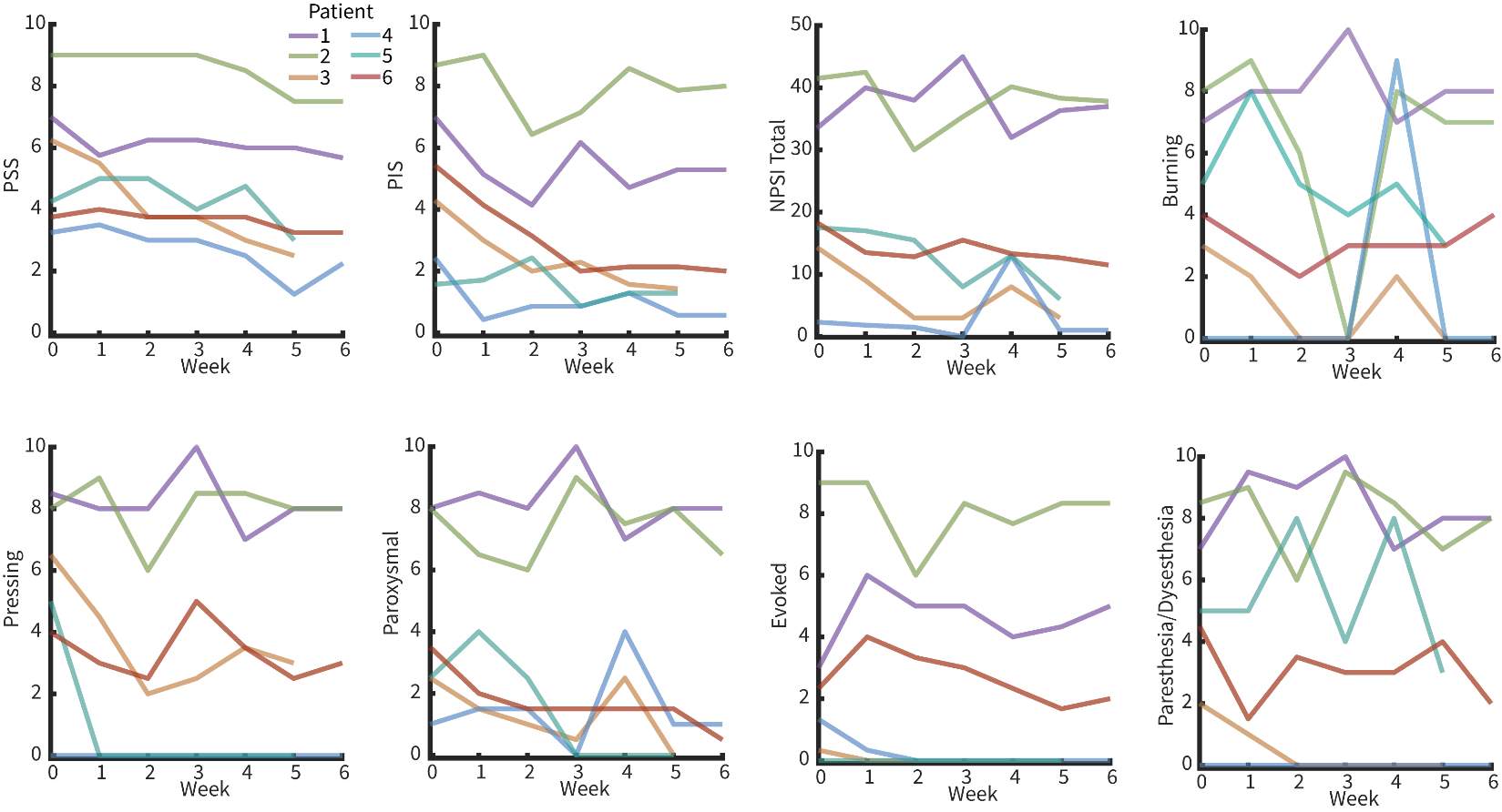


**Supplemental Figure 4. Weekly BPI and NPSI scores over the intervention period for individual patients.**

Clinical outcomes (NPSI, PSS, and PIS) over BCI therapy for each patient. Measures were taken via a patient-reported survey given at the end of each week. The first three graphs show PSS, PIS, and NPSI. The last five graphs show symptom scores for NPSI.


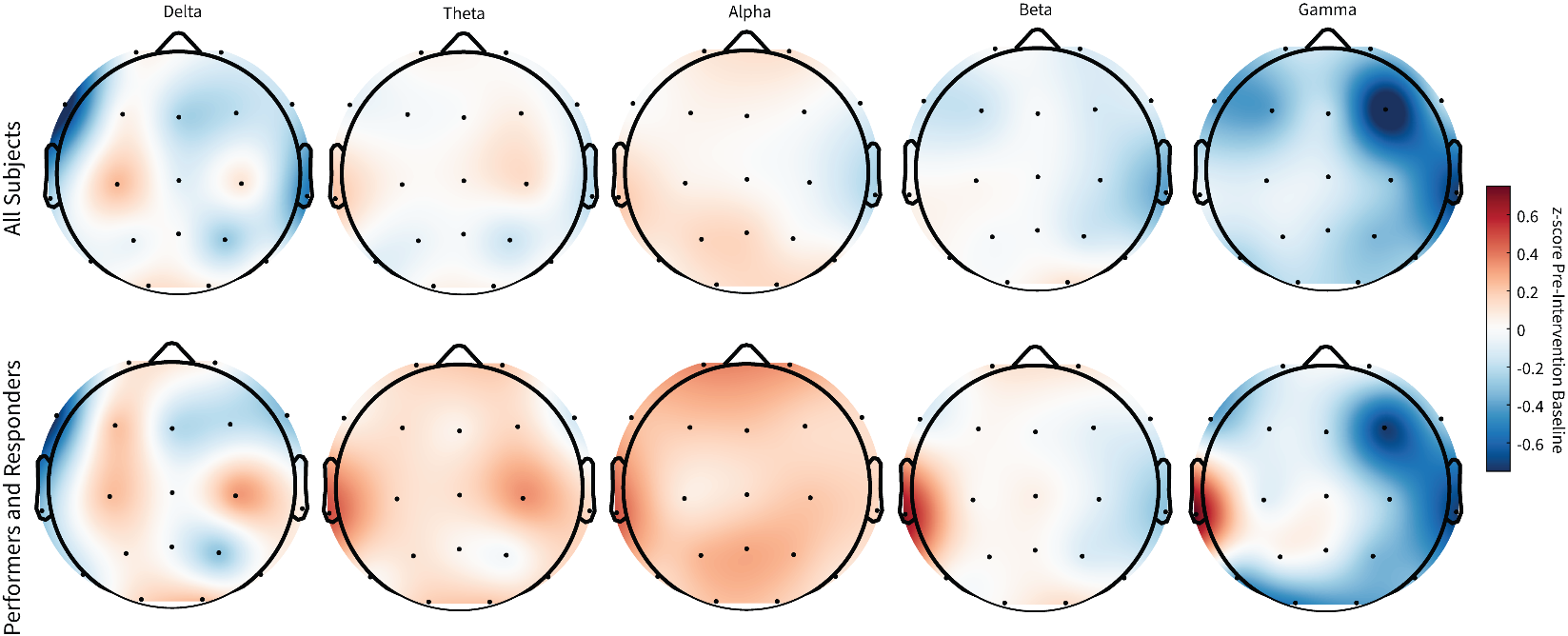


**Supplemental Figure 5. Topography plots of brain activity across patients before and after BCI intervention.**

Topography plots of baseline EEG activity for all frequency bands after BCI therapy for all subjects (Top) and for performers and VAS pain responders (Bottom) z-scored to pre-intervention baseline.


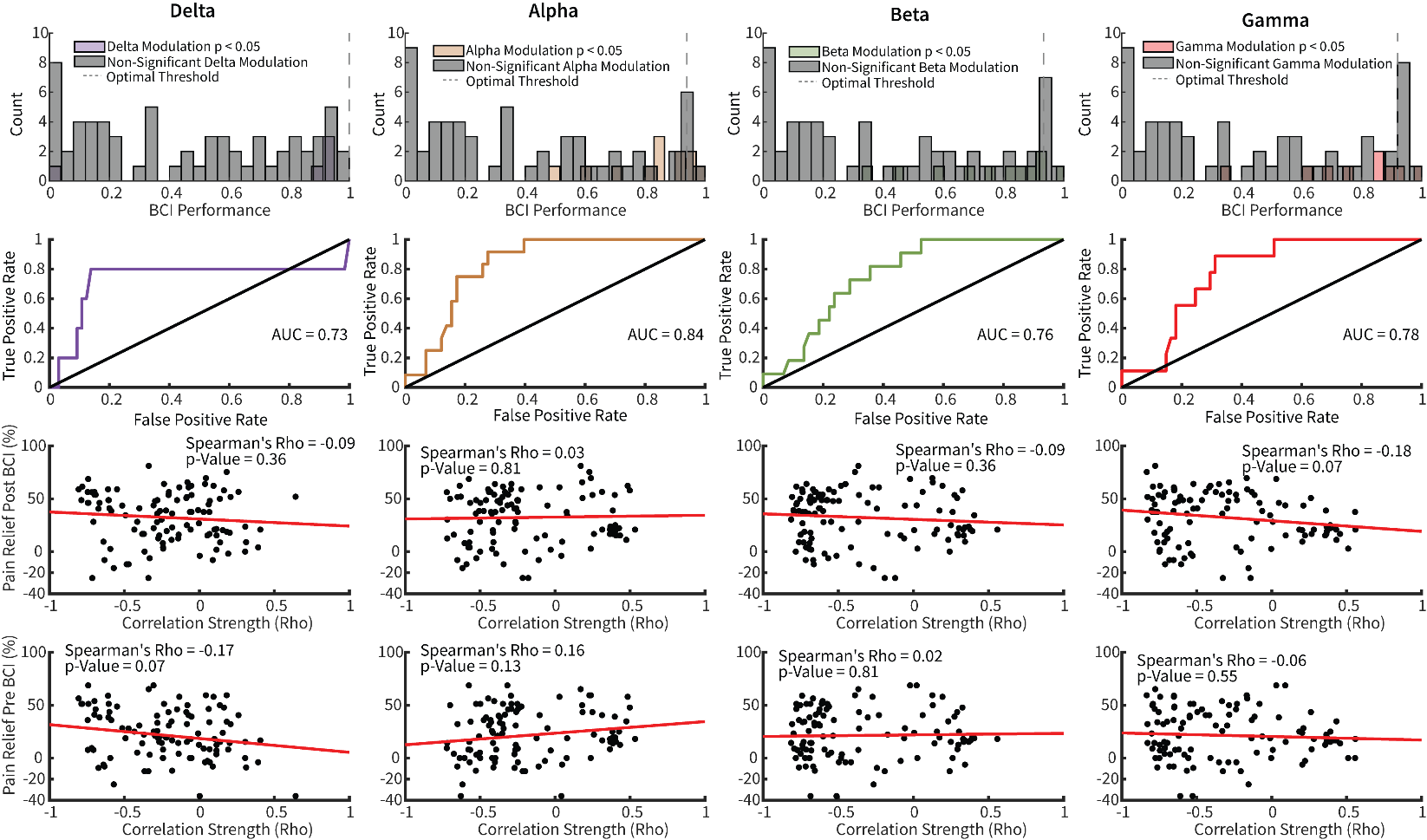


**Supplemental Figure 6. Correlation analysis of non-** θ **frequencies with pain relief.**

Secondary analysis of the relationship between (from left to right) delta, alpha, beta, and gamma modulation strength with BCI performance and pain relief. (First row) Distribution of all BCI sessions across all patients with and without significant modulation of respective frequency bands. The dotted line represents the optimal classification threshold calculated by ROC analysis. The shaded bars represent sessions with significant power modulation relative to the pre-intervention baseline (Spearman correlation, p < 0.05). (Second row) ROC curves demonstrating the true and false positive rates of correct classification of respective frequency modulation by performance value. (Third row) Relationship between respective frequency band modulation strength and pain relief using VAS obtained after each day’s BCI session (Spearman correlation). (Fourth row) Relationship between respective frequency band modulation strength and pain relief using VAS obtained before each day’s BCI session (Spearman correlation).

Supplementary Table 1. Patient quantitative sensory testing results.

|  | **CDT (C)** | | | **WDT (C)** | | **CPT (C)** | | **HPT(C)** | | **MDT (g)** | |
| --- | --- | --- | --- | --- | --- | --- | --- | --- | --- | --- | --- |
| **P** | Control | Affected | | Control | Affected | Control | Affected | Control | Affected | Control | Affected |
| 1 | 25.4 ±2.4 | 24.2 ±2.3 | | 44.2 ±1.2 | 38.3 ±1.5 | 0.0 ±0.0 | 0.0 ±0.0 | 46.1 ±3.5 | 47.0 ±0.6 | 4.6 ±0.3 | 4.2 ±0.3 |
| 2 | 30.0 ±0.3 | 28.3 ±0.9 | | 38.3 ±1.2 | 33.9 ±0.2 | 29.2 ±0.2 | 30.4 ±0.4 | 43.0 ±2.1 | 35.2 ±0.4 | 4.5 ±0.5 | 4.9 ±0.3 |
| 3 | 31.1 ±0.2 | 29.6 ±0.7 | | 32.8 ±0.7 | 33.7 ±0.3 | 0.0 ±0.0 | 0.0 ±0.0 | 44.2 ±1.6 | 48.1 ±1.2 | 2.2 ±0.5 | 2.0 ±0.4 |
| 4 | 24.3 ±1.0 | 29.1 ±1.1 | | 37.6 ±0.2 | 35.6 ±0.4 | 0.8 ±1.3 | 3.2 ±0.3 | 48.6 ±1.1 | 41.9 ±1.1 | 3.3 ±0.5 | 3.6 ±0.4 |
| 5 | 28.9 ±1.4 | 30.9 ±0.1 | | 35.4 ±0.9 | 36.2 ±0.6 | 8.8 ±7.7 | 24.0 ±1.8 | 44.4 ±5.1 | 45.4 ±1.3 | 4.0 ±0.1 | 3.7 ±0.6 |
| 6 | 30.3 ±0.3 | 30.4 ±0.2 | | 37.2 ±1.7 | 40.1 ±5.6 | 23.7 ±6.6 | 15.0 ±1.2 | 47.8 ±2.2 | 45.9 ±1.1 | 3.2 ±0.4 | 3.7 ±0.1 |
|  | **MPT(mN)** | | | **VDT (kHz)** | | **WUR** | | **PPT (kg/cm2)** | |  |  |
|  | Control | | Affected | Control | Affected | Control | Affected | Control | Affected |  |  |
| 1 | 138.7 ±112.4 | | 512.0 ±0.0 | 6.0 ±0.0 | 4.7 ±0.6 | 4.0 | 1.5 | 3.7 ±0.3 | 3.3 ±0.2 |  |  |
| 2 | 29.3 ±30.3 | | 24.0 ±13.9 | 4.0 ±1.0 | 2.7 ±0.3 | 0.8 | 1.0 | 1.1 ±0.1 | 1.1 ±0.1 |  |  |
| 3 | 512.0 ±0.0 | | 512.0 ±0.0 | 7.8 ±0.3 | 8.0 ±0.0 | 0.0 | 1.0 | 2.2 ±0.2 | 4.0 ±0.2 |  |  |
| 4 | 512.0 ±0.0 | | 512.0 ±0.0 | 6.7 ±0.3 | 6.8 ±0.3 | 1.0 | 2.0 | 3.2 ±0.5 | 1.6 ±0.4 |  |  |
| 5 | 512.0 ±0.0 | | 426.7 ±147.8 | 3.2 ±0.3 | 4.3 ±0.3 | 0.8 | 1.0 | 2.5 ±0.1 | 3.4 ±0.3 |  |  |
| 6 | 512.0 ±0.0 | | 45.3 ±32.3 | 8.0 ±0.0 | 8.0 ±0.0 | 0.8 | 1.0 | 2.2 ±0.3 | 2.3 ±0.3 |  |  |

Quantitative sensory testing was used to determine differential sensory profiles of each participant’s (P) affected (most painful) and unaffected (non-painful) regions. This was used to assess warm and cold detection (WDT and CDT) thresholds, heat and cold pain thresholds (HPT and CPT), mechanical detection and pain thresholds (MDT and MPT), presence of wind-up (enhanced temporal summation) to pinprick, vibration detection thresholds (VDT), and pressure pain threshold (PPT), and conditioned pain modulation (CPM). The Thermal Sensory Analyzer (TSA-II or PATHWAY platform; Medoc, Ramat Yishai, Israel) was used to determine WDT, CDT, HPT, and CPT. A set of standardized Semmes-Weinstein monofilaments (0.25, 0.5, 1, 2, 4, 8, 16, 32, 64, 128, and 256mN; North Coast, USA) was used to assess MDT for each participant’s affected and unaffected area. A set of calibrated blunt metal probes with applied force 8-512mN (MRC systems, Germany) was used to determine the MPT. Wind-up ratio (WUR) was assessed using a #6.10 von Frey filament (. 980mN) and calculated as the ratio between pain intensity elicited by a train of ten 1Hz stimuli versus a single stimulus value was calculated. Each participant’s frequency of vibration detection threshold was determined using a clinical tuning fork (64Hz, 8/8 scale). A handheld algometer (Wagner Instruments) was used to determine each participant’s PPT following continuous pressure application at a 0.5kg/sec rate. The test sites used for affected and control sites were along the same muscle.

Supplementary Table 2. Patient Conditioned Pain Modulation results. When tested for conditioned pain modulation (CPM) paradigm that assesses the efficiency of descending pain modulation, one patient had increased pain sensitivity during cold water conditioning, one patient had no change in pain sensitivity during cold water conditioning, and one patient could not tolerate the cold-water stimulus.

| **Patient** | **Preconditioning Evoked  Pain Intensity** | **CPM Pain Intensity with Conditioning** | **Delta** |
| --- | --- | --- | --- |
| 1 | 25.0 | 60.0 | 35.0 |
| 2 | N/A | N/A | N/A |
| 3 | 60.0 | 15.0 | -45.0 |
| 4 | 50.0 | 17.5 | -32.5 |
| 5 | 30.0 | 30.0 | 0.0 |
| 6 | 35.0 | 22.5 | -12.5 |

CPM testing includes the application of a “test” stimulus without conditioning, and a subsequent application of the same test stimulus with conditioning. The test stimulus is generated via a thermode applied at a volar forearm, with temperature ramping at 1°C/sec to a temperature that elicits pain intensity of 60 on 0-100 NRS in the individual. The conditioning stimulus includes the immersion of the contralateral hand up to the wrist to a thermostat-controlled water bath maintained at 12°C. The length of the conditioning stimulus is 60 seconds, and during the last 30 seconds of it the test stimulus is applied (at the contralateral forearm) twice, as described above. The difference between the intensity of pain stimulus with concomitant conditioning and between the intensity of pain stimulus without conditioning is the CPM magnitude. CPM<0 implies efficient descending pain modulation.

Supplementary Table 3. Total number of BCI trials for each patient on each session day, and across the entire intervention period. Days with a “0” indicate days when the participant was not scheduled for BCI training during the 30-day intervention period.

| **Patient** | **1** | **2** | **3** | **4** | **5** | **6** |
| --- | --- | --- | --- | --- | --- | --- |
| **Day 1** | 43 | 0 | 0 | 0 | 0 | 40 |
| **Day 2** | 61 | 0 | 45 | 38 | 0 | 65 |
| **Day 3** | 0 | 75 | 57 | 64 | 43 | 56 |
| **Day 4** | 67 | 59 | 60 | 63 | 0 | 56 |
| **Day 5** | 0 | 63 | 0 | 0 | 57 | 63 |
| **Day 6** | 0 | 82 | 0 | 53 | 64 | 52 |
| **Day 7** | 0 | 72 | 65 | 0 | 0 | 63 |
| **Day 8** | 80 | 71 | 71 | 0 | 64 | 57 |
| **Day 9** | 39 | 0 | 74 | 66 | 0 | 57 |
| **Day 10** | 42 | 0 | 0 | 74 | 34 | 68 |
| **Day 11** | 85 | 80 | 0 | 0 | 49 | 61 |
| **Day 12** | 0 | 93 | 80 | 60 | 47 | 66 |
| **Day 13** | 96 | 79 | 80 | 66 | 50 | 0 |
| **Day 14** | 80 | 0 | 87 | 0 | 0 | 72 |
| **Day 15** | 0 | 0 | 0 | 73 | 0 | 61 |
| **Day 16** | 83 | 87 | 0 | 77 | 48 | 60 |
| **Day 17** | 0 | 46 | 98 | 82 | 0 | 65 |
| **Day 18** | 0 | 89 | 0 | 69 | 44 | 62 |
| **Day 19** | 95 | 0 | 85 | 0 | 0 | 0 |
| **Day 20** | 82 | 0 | 91 | 0 | 49 | 72 |
| **Day 21** | 90 | 0 | 90 | 68 | 48 | 0 |
| **Day 22** | 84 | 0 | 78 | 60 | 0 | 64 |
| **Day 23** | 0 | 57 | 49 | 49 | 44 | 57 |
| **Day 24** | 80 | 64 | 0 | 0 | 0 | 65 |
| **Day 25** | 0 | 77 | 0 | 0 | 64 | 71 |
| **Day 26** | 0 | 76 | 0 | 68 | 75 | 77 |
| **Day 27** | 0 | 53 | 0 | 59 | 0 | 64 |
| **Day 28** | 85 | 51 | 0 | 0 | 35 | 59 |
| **Day 29** | 97 | 0 | 0 | 0 | 0 | 72 |
| **Day 30** | 100 | 0 | 0 | 0 | 67 | 66 |
| **Total** | 1389 | 1274 | 1110 | 1089 | 882 | 1691 |

Supplementary Table 4. Patient primary and secondary outcome measures. Bottom set contains scores for all sub-scores of NPSI.

|  | **VAS** | | **PSS** | | **PIS** | | **NPSI** | |  | |  |
| --- | --- | --- | --- | --- | --- | --- | --- | --- | --- | --- | --- |
| **Patient** | Pre | Post | Pre | Post | Pre | Post | Pre | Post |  | |  |
| 1 | 72.00 | 61.00 | 7.00 | 5.67 | 7.00 | 5.29 | 33.50 | 37.00 |  | |  |
| 2 | 56.00 | 38.00 | 9.00 | 7.50 | 8.67 | 8.00 | 41.50 | 37.83 |  | |  |
| 3 | 69.00 | 28.00 | 6.35 | 2.50 | 4.29 | 1.43 | 14.33 | 3.00 |  | |  |
| 4 | 32.00 | 41.00 | 3.25 | 2.25 | 2.43 | 0.57 | 2.33 | 1.00 |  | |  |
| 5 | 25.00 | 15.00 | 4.25 | 3.00 | 1.57 | 1.29 | 24.33 | 26.66 |  | |  |
| 6 | 39.00 | 17.00 | 3.75 | 3.25 | 5.43 | 2.00 | 12.33 | 13.33 |  | |  |
|  | **Burning** | | **Pressing** | | **Paroxysmal** | | **Evoked** | | **Paresthesia/dysesthesia** | |  |
|  | Pre | Post | Pre | Post | Pre | Post | Pre | Post | Pre | Post |  |
| 1 | 7.00 | 8.00 | 8.50 | 8.00 | 8.00 | 8.00 | 3.00 | 5.00 | 7.00 | 8.00 |  |
| 2 | 8.00 | 7.00 | 8.00 | 8.00 | 8.00 | 6.50 | 9.00 | 8.33 | 8.50 | 8.00 |  |
| 3 | 3.00 | 0.00 | 6.50 | 3.00 | 2.50 | 0.00 | 0.33 | 0.00 | 2.00 | 0.00 |  |
| 4 | 0.00 | 0.00 | 0.00 | 0.00 | 1.00 | 1.00 | 1.33 | 0.00 | 0.00 | 0.00 |  |
| 5 | 5.00 | 3.00 | 5.00 | 0.00 | 2.50 | 0.00 | 0.00 | 0.00 | 5.00 | 3.00 |  |
| 6 | 4.00 | 4.00 | 4.00 | 3.00 | 3.50 | 0.50 | 2.33 | 2.00 | 4.50 | 2.00 |  |

VAS = visual analog scale, PSS = pain severity score, PIS = pain interference score, NPSI = neuropathic pain symptom inventory score, Pre = pre-BCI intervention score, Post = post-BCI intervention score

Supplementary Table 5. Descriptive table showing methods and features of the continuous BCI used in this study.

| **Task Geometry:** |  |
| --- | --- |
| 1. Degrees of freedom: | 1-dimensional cursor with vertical movement (up and down) driven by the direction (increase or decrease) and magnitude of a single feature (normalized F3 theta power) |
| 2. Size and starting position: | Target: Rectangular shape with vertices at (-1,1), (1,1), (1,.9), (-1,.9). Its position remains static throughout the experiment. |
|  | Cursor: Starts at the center of the screen (0,0) and moves either up or down. The radius is 50 pixels. |
| 3. Distances: | Calculations are based on center-to-center positioning, but target touching is determined edge-to-edge. |
| 4. Units for all dimensions: | Positioning is described using the normalized device coordinate system (percentage of workspace display) with (0,0) as the center of the screen. Size measurements are given in pixels. |
| **End Effector Behavior:** |  |
| 1. Control Timing: | Phases and durations are as follows:  - Baseline: 10000ms - Start cue: 1000ms - Stop cue: 3000ms - Buffer: 7000ms  The cursor is under user control only during the neurofeedback phase. It leaves user control (i.e. resets to the center) either when the cursor's edge touches the rectangle's edge, or after the neurofeedback phase. |
| 2. Method of selecting targets: | There is only one target with a fixed position. |
| 3. Behavior of other targets: | N/A |
| 4. Speed gain settings: | Determined by the signal processing filter output which is then z-scored to the baseline distribution of signal processing outputs. |
| **Feedback Characteristics:** |  |
| 1. Form of online feedback: | Cursor movement and vibrotactile feedback |
| 2. Latency: | 50-60ms |
| 3. Feedback mode: | Online |

**SUPPLEMENTAL REFERENCES**

1. Finnerup, N. B., Sørensen, L., Biering-Sørensen, F., Johannesen, I. L. & Jensen, T. S. Segmental hypersensitivity and spinothalamic function in spinal cord injury pain. *Exp. Neurol.* **207**, 139–149 (2007).

2. Rolke, R. *et al.* Quantitative sensory testing in the German Research Network on Neuropathic Pain (DFNS): Standardized protocol and reference values. *PAIN* **123**, 231–243 (2006).

3. Baumgärtner, U., Magerl, W., Klein, T., Hopf, H. C. & Treede, R. D. Neurogenic hyperalgesia versus painful hypoalgesia: two distinct mechanisms of neuropathic pain. *Pain* **96**, 141–151 (2002).

4. Yarnitsky, D., Sprecher, E., Zaslansky, R. & Hemli, J. A. Heat pain thresholds: normative data and repeatability. *Pain* **60**, 329–332 (1995).

5. Fruhstorfer, H., Lindblom, U. & Schmidt, W. C. Method for quantitative estimation of thermal thresholds in patients. *J. Neurol. Neurosurg. Psychiatry* **39**, 1071–1075 (1976).

6. Magerl, W., Wilk, S. H. & Treede, R. D. Secondary hyperalgesia and perceptual wind-up following intradermal injection of capsaicin in humans. *Pain* **74**, 257–268 (1998).

7. Goldberg, J. M. & Lindblom, U. Standardised method of determining vibratory perception thresholds for diagnosis and screening in neurological investigation. *J. Neurol. Neurosurg. Psychiatry* **42**, 793–803 (1979).

8. Fagius, J. & Wahren, L. K. Variability of sensory threshold determination in clinical use. *J. Neurol. Sci.* **51**, 11–27 (1981).

9. Schacter, D. L. EEG theta waves and psychological phenomena: a review and analysis. *Biol. Psychol.* **5**, 47–82 (1977).

10. Inanaga, K. Frontal midline theta rhythm and mental activity. *Psychiatry Clin. Neurosci.* **52**, 555–566 (1998).

11. Gärtner, M., Grimm, S. & Bajbouj, M. Frontal midline theta oscillations during mental arithmetic: effects of stress. *Front. Behav. Neurosci.* **9**, 96 (2015).

12. Bazanova, O. M. & Vernon, D. Interpreting EEG alpha activity. *Neurosci. Biobehav. Rev.* **44**, 94–110 (2014).

13. Klimesch, W., Doppelmayr, M., Russegger, H., Pachinger, T. & Schwaiger, J. Induced alpha band power changes in the human EEG and attention. *Neurosci. Lett.* **244**, 73–76 (1998).
